# The stability and composition of the gut and skin microbiota of Atlantic salmon throughout the yolk sac stage

**DOI:** 10.3389/fmicb.2023.1177972

**Published:** 2023-07-06

**Authors:** Alexander W. Fiedler, Martha K. R. Drågen, Eirik D. Lorentsen, Olav Vadstein, Ingrid Bakke

**Affiliations:** Department of Biotechnology and Food Science, Norwegian University of Science and Technology, Trondheim, Norway

**Keywords:** Atlantic salmon, initial colonization, microbiota, yolk sac fry, 16s sequencing

## Abstract

The bacterial colonization of newly hatched fish is important for the larval development and health. Still, little is known about the ontogeny of the early microbiota of fish. Here, we conducted two independent experiments with yolk sac fry of Atlantic salmon that were (1) either reared conventionally, with the eggs as the only source for bacteria (egg-derived microbiota; EDM) or (2) hatched germ-free and re-colonized using lake water (lake-derived microbiota; LDM). First, we characterized the gut and skin microbiota at 6, 9, and 13 weeks post hatching based on extracted RNA. In the second experiment, we exposed fry to high doses of either a fish pathogen or a commensal bacterial isolate and sampled the microbiota based on extracted DNA. The fish microbiota differed strongly between EDM and LDM treatments. The phyla Proteobacteria, Bacteroidetes, and Actinobacteria dominated the fry microbiota, which was found temporarily dynamic. Interestingly, the microbiota of EDM fry was more stable, both between replicate rearing flasks, and over time. Although similar, the skin and gut microbiota started to differentiate during the yolk sac stage, several weeks before the yolk was consumed. Addition of high doses of bacterial isolates to fish flasks had only minor effects on the microbiota.

## 1. Introduction

All naturally living animals harbor a complex community of microorganisms, termed the animal’s microbiota. The microbiota as assemblages of commensal and pathogenic bacteria have mainly been studied in mammalian species like mice and humans, and it has been shown that the microbiota serves its host in a multitude of ways, e.g., by providing nutrients, protecting against pathogens and enabling a proper development (Lynch and Hsiao, 2019; Zheng et al., 2020). The early bacterial colonization is crucial for the host’s later development, influencing all aspects of adult life, like immune responses (Sevelsted et al., 2015), cognitive functions (Carlson et al., 2021), and nutrition (Huh et al., 2012).

These mechanisms are conserved between fish and mammals (Rawls et al., 2004; Phelps et al., 2017; Legrand et al., 2020; Borges et al., 2021). Fish live in close contact with bacteria in the surrounding water, which leads to an intimate relationship between fish and their surrounding bacteria. Fish therefore need strong barriers to protect themselves against unwanted microbes. The mucosal surfaces that cover the fish act as a selective barrier with antagonistic properties against pathogens by providing nutrients and colonization space for the surrounding bacteria (Merrifield and Rodiles, 2015; Gomez and Primm, 2021). These mucosal surfaces on both skin and digestive tract are therefore the primary interaction site between the fish, its microbiota, and the microbes of the surrounding water (Li et al., 2019).

Generally, fish are microbe-free when they are in their eggs and immediately get colonized by the water microbes after hatching, making bacteria in the water an important source of the fish’s microbiome at this stage (Llewellyn et al., 2014). This contrasts with mammals, where the initial microbiota mainly originates from the mother’s microbiome (Ferretti et al., 2018). Generally, the skin of fish is colonized by bacteria immediately after hatching, and consensus is that the gut is colonized after the opening of the mouth (Reitan et al., 1998; Lokesh et al., 2019; Nikouli et al., 2019). Detailed studies of the early fish microbiota have been conducted in only a few species, and with a focus on the gut (reviewed by Borges et al., 2021). These few studies indicate that the initial fish gut bacterial community is dominated by Proteobacteria, Bacteroidetes, Firmicutes, and Actinomycetes and is increasing in diversity from early larval stages until the juvenile stage (Borges et al., 2021). Lokesh et al. (2019) investigated the ontogeny of the microbiota of Atlantic salmon and found that Proteobacteria were dominating the gut microbiota during the weeks before feeding. The onset of feeding seems to have a great influence on the composition of the gut microbiota (Ingerslev et al., 2014a,b; Michl et al., 2019). However, a well-established gut microbial community is probably already present before the fish start exogenous feeding (Ingerslev et al., 2014a,b; Sun et al., 2015; Stephens et al., 2016; Califano et al., 2017; Nikouli et al., 2019; Wilkes Walburn et al., 2019). Which factors influence these early microbial communities is, however, not thoroughly examined.

Further, not much research has been done on comparing the larval gut and skin microbiota and how they are assembled and interact (Dodd et al., 2020; Gomez and Primm, 2021). For adult fish, both the skin and gut microbiota are influenced by both abiotic factors (e.g., water temperature, salinity and diet) and biotic factors (e.g., sex, genetic background, and developmental stage) (Bakke et al., 2015; Dehler et al., 2017; Legrand et al., 2020). Viral and bacterial infections also influence the microbial community structures in the fish (Ingerslev et al., 2014a,b; Reid et al., 2017; Bozzi et al., 2021). It is further assumed that certain bacterial groups are selected for at the mucosal surfaces of the fish (Reitan et al., 1998; Lokesh et al., 2019).

Understanding the factors that affect the community assembly in developing fish is important as it could be used to steer against the presence of pathogenic and opportunistic bacteria in aquaculture systems, and thereby counteract negative fish-microbe interactions (Verschuere et al., 2000; Vadstein et al., 2018). This is especially important in the early life stages, when the immune system is not fully developed (Zapata et al., 2006) and the fish are explicitly vulnerable (Vadstein et al., 2013). The early life stages therefore generally represent a bottleneck in aquaculture (Sifa and Mathias, 1987). It has further been suggested that the fish microbiota plays a crucial role in protecting especially fish in their early life stages against pathogens (Liu et al., 2014; Pérez-Pascual et al., 2021; Stressmann et al., 2021). The early life stages of fish are therefore especially interesting for treatments to promote positive host–microbe interactions to reduce mortality and sickness. However, this requires more fundamental knowledge of mechanisms involved in community assembly of early life stages.

Atlantic salmon (*Salmo salar*) is an important aquaculture species, with more than 2.4 million tonnes being produced per year (FAO, 2020). This species has a long yolk sac stage of around 500 day-degrees, and recently a protocol for raising germ-free salmon has been developed (Gomez de la Torre Canny et al., 2022). Thus, germ-free or gnotobiotic Atlantic salmon can be kept in their yolk sac stage for as long as 13 weeks at 6°C without feeding. This system is therefore an ideal model system for studying the initial colonization of a host and allows for complete manipulation of the colonizing bacterial communities. By using this experimental design, we recently showed that colonizing newly hatched salmon with fish distinct aquatic microbial communities resulted in distinct fish microbiota, which again influenced the skin mucosa, the somatic growth, and the utilization of the yolk (Gomez de la Torre Canny et al., 2022).

In this study, we used the gnotobiotic Atlantic salmon model system to investigate the initial bacterial colonization and the development of the gut and skin microbiota of Atlantic salmon throughout the yolk sac stage. We aimed to assess the influence of the composition of the bacterial source community present at hatching on the development of both the gut and skin microbiota. We hatched fish under germ-free conditions and exposed them to either their egg microbiota or to a lake water microbiota. Furthermore, we examined the potential for manipulating the early larval bacterial communities by exposing the fish to high concentrations of both a presumptive pathogenic (*Yersinia ruckeri* 06059) and a putative commensal bacterial strain (*Janthinobacterium* sp. 3.108). Finally, we compared skin and gut microbiota to the microbiota of the rearing water. Characterization of the host microbiota was done by extracting RNA (Exp.1) or DNA (Exp.2) from gut and skin samples (and water samples for Exp.2) and sequencing the v3 + v4 hypervariable region of the 16S rRNA (gene) using the Illumina platform. The microbiota analysis of Exp.1 was based on extracted RNA instead of DNA as the original intend with these samples was to investigate gene expression in the fish. Here, we used the extracted RNA to characterize the microbiota of the yolk sac fry.

## 2. Materials and methods

### 2.1. Experimental design

Two independent fish experiments were conducted from October 2019 to January 2020 (Exp.1) and from February to April 2020 (Exp.2).

For both experiments, Atlantic salmon yolk sac fry were raised under two microbial conditions: (1) Fish were raised under conventional microbial conditions, i.e., the eggs were not sterilized after arrival to the laboratory. However, they were hatched and reared in a sterile freshwater medium so that the only source of bacteria for colonization after hatching was bacteria originating from their eggs (egg-derived microbiota, EDM). (2) Alternatively, eggs were hatched under germ-free conditions and then exposed to bacteria by adding untreated lake water to the sterile rearing flasks (lake-derived microbiota, LDM). In the following, the terms “EDM flasks” and “LDM flasks” are used to refer to these two experimental groups, and the terms “EDM samples” and “LDM samples” are used to refer to samples taken from EDM and LDM rearing flasks, respectively.

For Exp.1, samples were collected during the experiment described in Figure 4A in Gomez de la Torre Canny et al. (2022) from conventionally raised fish (corresponding to the EDM experimental group in the present study) and conventionalized fish (corresponding to the LDM experimental group in the present study). Gomez de la Torre Canny et al. (2022) included the analysis of the skin and gut at 13 weeks post hatching (wph). Here, we extended the analyses of the gut and skin microbiota at 13 wph and also included samples taken at 6 and 9 wph from two replicate flasks per sampling time. Characterization of microbial communities was performed by Illumina sequencing of the v3 + v4 16S rRNA amplicons, based on RNA extracts from gut and skin samples (for details, see below). Total RNA was extracted from fish samples in Exp.1 instead of DNA, because the samples were originally planned to be used to study gene expression in the fish by qPCR. Here, we used the extracted RNA to analyze the microbial communities.

Exp.2 was originally designed as a challenge experiment, with *Y. ruckeri* as the pathogen, and the commencal *Janthinobacterium* sp. 3.108 as a control, representing a non-pathogenic bacterium. The design originally included both germ-free fish, and colonized fish (EDM and LDM). However, the *Y. ruckeri* strain did not induce mortality in the fish, and we were therefore not able to investigate the potential protective role of the fish microbiota in pathogenic infection. Here, we used the fish and water samples that were collected to examine the effect on the fish and water microbiota of the exposure to high doses of *Y. ruckeri* and *J*. sp. 3.108. The experiment had a factorial design, with source bacterial community (EDM vs. LDM) and addition of high quantities of two bacterial isolates (added vs. not added) as the two factors. Exp.2 included nine EDM and nine LDM flasks. At 6 wph, either the fish pathogen *Yersinia ruckeri* 06059 or the fish commensal *Janthinobacterium* sp. 3.108 was added to three replicate flasks for both EDM and LDM, whereas three flasks were left untreated. This resulted in six experimental groups. Characterization of bacterial communities by 16S rRNA gene amplicon sequencing was based on total DNA extracts from gut, skin and water samples taken at 8 wph.

### 2.2. Fish husbandry

In general, the derivation of Atlantic salmon eggs and their husbandry as described in Gomez de la Torre Canny et al. (2022) was followed for Atlantic salmon husbandry. Briefly, salmon eggs were obtained at around 80% developmental status from AquaGen AS (Hemne, Norway), transferred to a dark room, and kept at a constant water temperature of 5.8 ± 0.3°C. The eggs were placed in petri dishes (13.5 cm Ø) at a density of 100 eggs/dish and covered with Salmon Gnotobiotic Medium (SGM). SGM contained 0.5 mM MgSO_4_, 0.054 mM KCl, 0.349 mM CaSO_4_ and 1.143 mM NaHCO_3_ dissolved in MilliQ water and was sterilized by autoclaving prior to use (121°C for 20 min). One day after arrival, the fish eggs were split into two groups. One group was surface-sterilized to obtain germ-free fish [for generating conventionalized fry; LDM group, corresponding to CVZ in the study by Gomez de la Torre Canny et al. (2022)], whereas the other group was not treated [for generating conventionally reared fry; EDM group, corresponding to CVR in the study by Gomez de la Torre Canny et al. (2022)]. Two days after arrival, all eggs were distributed into 250 ml cell culture flasks with vented caps and covered with 100 ml sterile SGM (17 eggs per flask). The eggs, and, after hatching, the fish, were reared in these flasks for the rest of the experiment. To maintain good water quality, 60% of the SGM in the fish flasks was exchanged three times a week and replaced with sterile SGM. Fish mortality was checked regularly, and dead fish were removed. For sampling and at the end of the experiment, fish were euthanized by a lethal dose of tricaine [5.2 g tricaine (20 mM final concentration) in 27.3 ml 1 M Tris buffer (pH 9), ad 1 L with SGM, sterilized by filtration through a 0.2 μm filter].

### 2.3. Sterilization of fish eggs and reintroduction of bacteria (conventionalization)

The sterilization procedure described by Gomez de la Torre Canny et al. (2022) was followed. Eggs were surface-sterilized 24 h after arrival at our laboratory. The eggs were submerged in an antibiotic cocktail (10 mgl^−1^ Rifampicin, 10 mgl^−1^, Erythromycin, 10 mgl^−1^ Kanamycin, 100 mgl^−1^ Ampicillin, 250 μg/l^−1^ Amphotericin B, 150 mgl^−1^ Penicillin, and 75 mgl^−1^ Oxolinic acid) and incubated at 6°C for 24 h. Afterwards, groups of 17 eggs were incubated in a Buffodine® solution (FishTech AS) containing 50 mgl^−1^ available iodine for 30 min, washed four times in 50 ml SGM and were then transferred into 250 ml cell-culture flasks with vented caps containing 100 ml SGM. A sterility check was performed on the hatching day (hatching day defined as the day when 80% of all eggs have hatched) and regularly throughout the experiment by inoculating four different liquid media (Brain Heart Infusion, Glucose Yeast Broth, Sabourad-Dextrose Broth and Nutrient Broth) and Tryptic Soy Agar plates with 100 μl rearing water. The liquid media and TSA plates were incubated at room temperature for up to 3 weeks. If bacterial growth was observed in one of the media, the fish flask was considered contaminated and was removed from the experiment. One week after hatching, the axenic fish were conventionalized by removing 60 ml rearing water and adding 60 ml water from the lake Jonsvatnet (Trondheim, Norway). The water from lake Jonsvatnet was untreated and taken from a depth of 50 m in October 2019 (Exp.1) and March 2020 (Exp.2), respectively.

### 2.4. Isolation of *Janthinobacterium* sp. 3.108

*Janthinobacterium* sp. strain 3.108 was isolated from the skin of healthy Atlantic salmon fry in a commercial flow-through-system as follows: skin was scraped off both sides of an individual under aseptic conditions. The skin mucus was collected in a cryotube, added 500 μl glycerol (50%), snap-frozen on dry ice, and transported back to the laboratory. The sample was added Maximum Recovery Diluent (MRD), thawed and homogenized using a glass rod (MRD added step-wise to a total of 1 ml) and finally vortexed. The homogenate was serial diluted (1:10) in MRD and streaked on Plate Count Agar plates (PCA; 5 g tryptone, 2.5 g yeast extract, 1 g glucose and 12 g bacteriological agar per l). Single colonies were picked and resuspended in 50 μl MRD, serial diluted in MRD and streaked again on PCA plates. This was repeated two more times to ensure that the picked colony represented a single bacterial isolate. The isolate was taxonomically assigned by PCR amplification of the 16S rRNA gene, followed by Sanger sequencing. PCR was performed using the primers Eub8F (5′-AGAGTTTGATCMTGGCTCAG-3′) and 1492R (5’-TACGGYTACCTTGTTACGACTT-3′). The PCR reactions were run for 35 cycles (98°C 15 s, 55°C 20 s, and 72°C 20 s) with 0.2 mM of each dNTP, 0.3 μM of each primer, and Phusion Hot Start II DNA polymerase and reaction buffer (Thermo Scientific). As template, we used 1 μl of a lysate generated by boiling a colony of the relevant isolate for 10 min. The resulting PCR product was purified using the QIAquick® PCR Purification Kit (Qiagen) as described by the manufacturers. The purified PCR product (5 μl) were mixed with 5 μl sequencing primer (5 mM) and sent to Eurofins Genomics for Sanger sequencing. Three sequencing primers were applied: Eub8F, 1492R, and 805R (5′-ATTACCGCGGCTGCTGG-3′). For the resulting sequences, regions of poor quality in the 5′- and 3′-ends, as well as primer sequences, were trimmed off, and the sequences were assembled. The resulting sequence is provided in Supplementary Figure S7.

### 2.5. Bacterial exposure in Exp.2

At 5 wph, the number of fish was adjusted to 10 individuals per flask and the water temperature was gradually increased to 14.0 ± 0.1°C over the course of 7 days. Two bacterial isolates were used in this experiment: *Y. ruckeri* 06059 and *Janthinobacterium* sp. 3.108 (described above). A virulent strain of *Yersinia ruckeri* (strain 06059; Serotype O1) that was isolated from Atlantic salmon in the UK in 2006 was kindly provided by Tim Wallis (Ridgeway Biologicals Ltd., UK; Haig et al., 2011). At 6 wph, three EDM and LDM flasks were added *Y. ruckeri* and three *J*. sp. 3.108. Three EDM and three LDM flasks served as untreated control. *Y. ruckeri* and *J*. sp. 3.108 were grown in liquid TSB medium overnight at room temperature in an orbital shaker at 120 rpm under aerobic conditions and harvested at an OD_600_ of app. 1. One ml culture was centrifuged at 13.000xg for 1 min to obtain a bacterial pellet. The pellet was washed with SGM once, before it was resuspended in 1 ml of SGM and added to the fish flasks. This resulted in a theoretical final concentration of app. 10^7^ CFUml^−1^ of the respective strains in the fish flasks. After addition of bacteria, the fish were reared at 14°C for 2 weeks (until 8 wph) and then sampled.

### 2.6. Sampling

For Exp.1, three fish were sampled from each of two replicate flasks for both EDM and LDM fish per timepoint (12 fish sampled at 6, 9, and 13 wph), resulting in a total of 36 fish sampled. The flasks were removed from the experiment after sampling. Sampling of gut and skin at 13 wph in Exp.1 is described by Gomez de la Torre Canny et al. (2022) and samples from 6 and 9 wph of Exp.1 were prepared the same way. In brief, individual fish were transferred to individual wells of a 12-well plate prefilled with sterile SGM. The SGM was replaced with sterile tricaine solution for euthanization and each fry was rinsed three times with sterile SGM. Excess SGM was removed and fish were individually dissected in sterile petri dishes. Using sterile forceps, the yolk sac was removed and discarded. The gut was dissected out of the fish by pulling it out from esophagus to anus and was placed in screw-cap centrifuge tubes prefilled with 200 μl 1.4 mm zirconium beads and TRIzol (0.5 ml TRIzol for gut samples, 0.75 ml for skin samples from 6 and 9 wph and 1 ml TRIzol for skin samples from 13 wph). For samples from 6 and 9 wph, the remainder of the fish was used as an approximation for a skin sample, since the skin mucosa could not be dissected off the fish at these early stages, while for samples taken at 13 wph the skin was dissected off the fish.

In Exp.2, three fish were sampled at 8 wph from three flasks each of both EDM and LDM flasks for the two bacteria-treated groups and the untreated control. In addition, a water sample was taken from each flask, resulting in a total of 54 fish samples and 18 water samples. Fish samples were prepared by replacing the rearing water of the sampled fish flasks with sterile tricaine solution. After euthanisation of the fish, individual fish were transferred to individual wells of a 6-well petri dish prefilled with sterile SGM. For rinsing, each individual was transferred to a new well prefilled with sterile SGM. The fish were removed from the wells using sterile forceps and excessive SGM was removed using Kimtech-Wipes, without the fish touching the wipes. Each fish was transferred to a sterile petri dish and was dissected under a stereoscope. Using sterile forceps, the yolk sac was removed and discarded and the gut was dissected from the fish by pulling it from anus to esophagus. The rest of the fish was used as skin sample. Gut and skin samples were each transferred into separate 2-ml empty sterile screw-cap centrifuge tubes. Water samples were taken by filtrating 45 ml of fish rearing water through a 0.2 μm filter (STERIVEX™, Millipore) and placing the filter in an empty sterile 2 ml screw-cap tube. All samples from both experiments were snap frozen in liquid nitrogen and stored at −80°C until DNA/RNA extraction.

### 2.7. DNA and RNA extraction and cDNA synthesis

For Exp.1, total RNA was extracted and cDNA synthesized as previously described (Gomez de la Torre Canny et al., 2022). In brief, gut and skin samples were homogenized and total RNA was extracted using the Purelink™ RNA Mini Kit (Invitrogen™), then treated with DNase (On-Column Purelink DNase Treatment; Invitrogen), and immediately frozen at – 80°C. The iScript™ cDNA Synthesis kit (Bio-Rad) was used for cDNA synthesis with 800 ng DNase treated RNA as template, following the manufacturer’s instructions.

For samples collected in Exp.2, DNA was extracted from skin, gut and water samples using a KingFisher Flex instrument with the ZymoBIOMICS™ 96 MagBead DNA kit. First, all samples were homogenized and lysed in 750 μl lysis buffer from the kit by vortexing them horizontally in 2 ml screwcap tubes with 1.4 mm Zirconium beads for 45 min. DNA was extracted from 300 μl lysate following the kit’s protocol for the KingFisher Flex (50 μl DNAse-free water was used for elution) and samples were frozen at −20°C until examination. For a few samples we could not generate 16S rRNA gene amplicons, here, the DNA extraction was repeated using the remaining 400 μl of the lysate.

### 2.8. Amplification of the v3-v4 region of the 16S rRNA gene

Two amplicon libraries were prepared, one for samples from Exp.1 and one for samples from Exp.2. For DNA extracts from Exp.2, the v3 + v4 region of the 16S rRNA gene was amplified using the primers Ill-338F (5′-TCGTCGGCAGCGTCAGATGTGTATAAGAGACAGNNNN**ACTCCTACGGGWGGCAGCAG** -3′) and Ill-805R (5’-GTCTCGTGGGCTCGGAGATGTGTATAAGAGACAGNNNN**GACTACNVGGGTATCTAAKCC** -3′), with the target sequences shown in bold (Nordgard et al., 2017). For the cDNA representing total RNA from the samples from Exp.1, we had problems with co-amplification of host DNA, and therefore designed a new forward primer that had lower similarity to the *Salmo salar* 18S rRNA gene (Ill-329F: 5′-TCGTCGGCAGCGTCAGATGTGTATAAGAGACAGNNNN**ACKGNCCWDACWCCTACGGG**-3′; Gomez de la Torre Canny et al., 2022). The same reverse primer as for Exp.2 was used for Exp.1.

The PCRs were performed in 25 μl total reaction volume with either 1 μl cDNA extracts (Exp.1) or 2 μl of 1:10 diluted DNA extracts (Exp.2) as templates. Each PCR reaction contained 0.3 μM of each primer (0.15 μM for Exp.2) and 0.25 μM of each dNTP as well as 0.4 U Phusion hot start polymerase and the respective buffer from Thermo Scientific. The PCRs were run with the following temperature cycling conditions: an initial denaturation step at 98°C for 60 s followed by 38–40 cycles (33 cycles for water samples) of 98°C for 15 s, 58°C for 20 s (55°C for the second experiment) and 72°C for 20 s. The final elongation step was 72°C for 5 min before the samples were cooled to 10°C. PCR products were evaluated by electrophoresis on 1.5% agarose gels containing 50 μM GelRed (Biotium) for 1 h at 110 V.

### 2.9. Amplicon library preparation and Illumina sequencing

PCR products of expected size and quantity were normalized using Sequal Prep™ Normalization plates (96 wells, Invitrogen) before they were indexed using the Nextera® XT Index Kit v2 Set A in a second round of PCR. Indexing PCR consisted of 2.5 μl normalized and purified PCR product as template, 2.5 μl of both indexing primers, 0.25 μM of each dNTP, 0.5 mM MgCl_2_ (in addition to MgCl_2_ contained in the buffer) as well as 0.4 U Phusion hot start polymerase and the respective buffer from Thermo Scientific in a total reaction volume of 25 μl. The indexing PCR was run with an annealing temperature of 58°C and 10 cycles, the other cycling conditions were as described above. The indexed PCR products were normalized using the Sequal Prep Normalization kit and then pooled and up-concentrated using an Amicon® Ultra 0.5 ml centrifugal filter (30 K membrane, Merck Millipore). The quality of the DNA of the amplicon libraries was determined using a NanoDrop™ One Microvolume Spectrophotometer (Thermo Scientific™). For Exp.1, the amplicon library included 93 samples, whereas for Exp.2 the amplicon library included 96 samples (both libraries included few samples not relevant for this study). The samples were sent to the Norwegian Sequencing Center using one run on a MiSeq v3 instrument for each amplicon library with 300 paired ends. The sequencing data was deposited at the European Nucleotide Archive (ERS14440101-ERS14440192).

### 2.10. Analysis of the Illumina sequencing data

The USEARCH pipeline (v.11) (Edgar, 2010) was used to process the data obtained from Illumina sequencing. For Exp.1, all data were processed together as described in Gomez de la Torre Canny et al. (2022), while the data obtained from Exp.2 were processed together using the same pipeline. In brief, the paired reads were merged, and primer sequences trimmed off using the Fastq_mergepairs command with a minimal length of 390 bp. The merged sequences were quality-filtered using the Fastq-filter function with the default error threshold value of 1. The reads were pooled, dereplicated and singleton reads removed. Zero-range OTUs (zOTUs, synonymous to amplicon sequence variants, ASVs) were generated using the Unoise3 command (Edgar, 2016b) with the default minimum abundance threshold of 8 reads in the total dataset. Taxonomical assignment of the ASVs was achieved using the Sintax command (Edgar, 2016a) with a confidence threshold of 0.8 and the ribosomal database project (RDP) reference dataset. RDP training set v16 was used for the data obtained from Exp.2 and training set v18 for the data obtained from Exp.1. A minor fraction of the reads was classified as eukaryotes and chloroplasts and were removed from the data set. A few ASVs that were highly abundant in negative controls for the DNA extraction, but less abundant in the samples, were considered to represent contaminating DNA associated with the DNA extraction kit and/or PCR reagents and were removed from the data sets. For Exp.2, ASV3 and ASV15 were combined to ASV3-15, since both ASVs corresponded to *Janthinobacterium* sp. 3.108, which has two highly similar 16S rRNA gene sequences, differing in only one base pair that corresponded to an ambiguous nucleotide position in the 16S rDNA sequence of *J*. sp. 3.108 (Supplementary Figure S7). After quality filtering, the 70 samples of Exp.1 contained a total of 1,562 ASVs and 4,777,641 reads (68,252 reads per sample on average). For Exp.2, the 91 samples consisted of 598 ASVs and 9,583,497 reads (105,312 reads per sample on average). The mean sequencing depth, as indicated by Chao-1 was 83.8% for Exp.1 and 83.4% for Exp.2 (Supplementary Tables S1, S2). The final ASV tables were normalized by scaling to 26,000 reads per sample (Exp.1) and 43,347 reads per sample (Exp.2), respectively. All statistical analyses were performed using the normalized ASV tables.

### 2.11. Statistical analysis

All statistical analyses were performed in R (v. 4.0.4)^1^ using the packages Phyloseq (v. 1.34.0) and Vegan (v. 2.5.7). α-diversities were calculated as Hill’s diversity numbers (Hill, 1973; Lucas et al., 2017) using the *renyi* function of vegan. The evenness was calculated by dividing Hill’s diversity of order 1 (exponential Shannon index) by Hill’s diversity of order 0 (richness). Ordination by principal coordinate analyses (PCoAs) were performed using the *ordinate* function from phyloseq for Bray–Curtis similarities, if not stated otherwise. For PCoAs based on weighted Unifrac analysis, phylogenetic trees were generated using the MEGA-X software. The trees were generated employing the maximum likelihood method using a Tamura-Nei model with 1,000 bootstrap replications. The trees were rooted by using the longest branch as root. To compare similarity in community composition between groups of samples, PERMANOVA analyses (Anderson, 2001) based on Bray–Curtis similarities (if not stated otherwise) were done using the *adonis*2 function from vegan by running it in 100 iterations with 999 permutations each and the mean value of p of the 100 iterations was reported (mathematically lowest possible value of *p* = 0.001). Whenever the sample size allowed it, PERMANOVAs were run as nested PERMANOVAs with “replicate flask” as sublevel. For statistical univariate data (e.g., α*-*diversity indices or abundance of certain ASVs), the data was checked for normality using the Shapiro–Wilk test (*shapiro.test* function). Generally, the data were not normally distributed and therefore a Mann–Whitney U test was used for data with two groups (*wilcox.test* function) and a Kruskal–Wallis test (Kruskal.test function) was used when more than two groups were compared. A significant Kruskal–Wallis test was followed by a Bonferroni-corrected Dunn test (*dunnTest* function).

## 3. Results

### 3.1. Hatching rate and survival of the fish

The hatchability of eggs in Exp.1 was very high and has already been reported in Gomez de la Torre Canny et al. (2022). The hatchability of Exp.2 was equally high, being >90% in both LDM and EDM flasks. For Exp.2, none of the fish died after addition of *Y. ruckeri* and two fish died after *J.* sp. 3.108 was added to their replicate flasks. One fish died in the untreated control group. The bacterial fish pathogen *Y. ruckeri* did therefore not induce mortality in the 6-week-old Atlantic salmons under the experimental conditions applied in this study.

### 3.2. The influence of the source microbiota on the gut and skin microbiota of Atlantic salmon larvae

**Figure 1:**
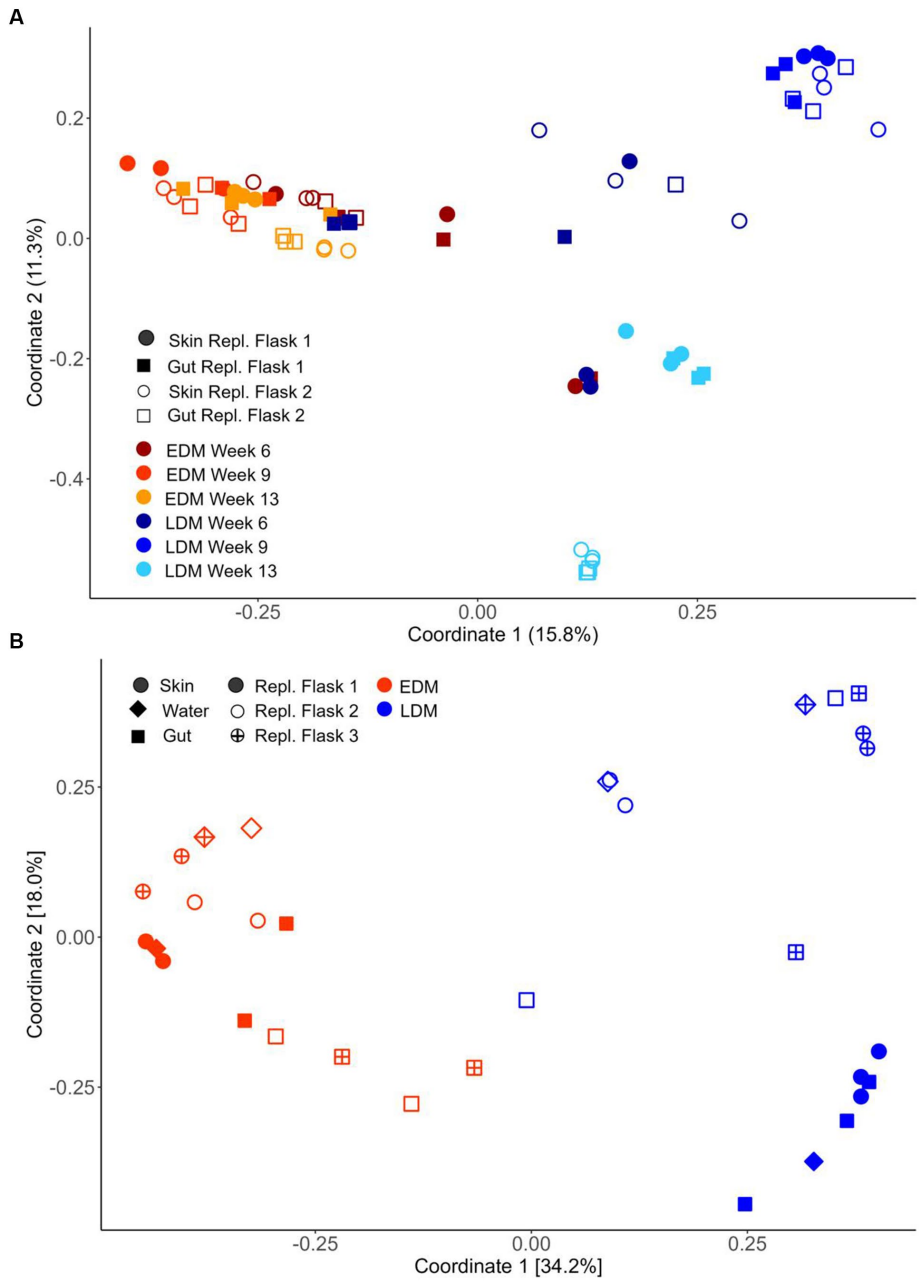
Ordination by PCoA based on Bray–Curtis similarities for skin, water (only Exp.2) and gut samples for groups receiving egg-derived (EDM) or lake-derived (LDM) microbiota. **(A)** Samples from Exp.1 based on 16S rRNA and **(B)** untreated samples from Exp.2 based on the 16S rRNA gene. For all samples of Exp.2, see Figure 6.

The fish in both experiments included in this study derived their bacterial communities from one of two source microbiota, either from their eggs (egg-derived microbiota, EDM, i.e., the eggs were not hatched germ-free, but in the presence of the microbiota associated with the eggs) or from lake water (lake-derived microbiota, LDM, lake water added to germ-free fry soon after hatching). All the bacteria in EDM flasks therefore originated from the fish eggs (EDM source microbiota) and all bacteria in the LDM flasks originated from the freshwater lake water (LDM source microbiota). Principal coordinate analysis (PCoA) based on the Bray–Curtis similarities for samples from Exp.1 showed that the fish microbiota differed considerably between EDM and LDM samples (Figure 1A). A nested PERMANOVA test with “replicate flask” as sublevel showed that the fish microbiota differed significantly between EDM and LDM samples at all sampling times (6, 9 and 13 wph; PERMANOVA, value of *p*s = 0.002, ≤0.001 and ≤0.001, respectively; gut and skin samples combined). Average Bray–Curtis similarities showed that the microbiota of the EDM and LDM became increasingly different with increasing age (Supplementary Figure S1).

Also for Exp.2, a PCoA corroborated this finding, and showed a clear separation of the fish microbiota between the EDM and LDM samples (Figure 1B). A nested PERMANOVA test with “replicate flask” as sublevel showed again that the fish microbiota differed significantly between LDM and EDM samples (value of *p* ≤ 0.001). Interestingly, the separation between the microbiota of the EDM and LDM fish was less prominent in a PCoA based on weighted UniFrac distances (Supplementary Figure S2). However, for Exp.1 the differences were still significant for samples from week 9 and 13 (nested PERMANOVA, *p* ≤ 0.001; gut and skin combined) but not for week 6 samples (*p* = 0.464). Also for Exp.2, the nested PERMANOVA showed that the difference between EDM and LDM microbiota was significantly different when UniFrac distances were used (*p* = 0.019). Altogether, these results show that the source microbiota had a major impact on the bacterial communities associated with the fish.

**Figure 2:**
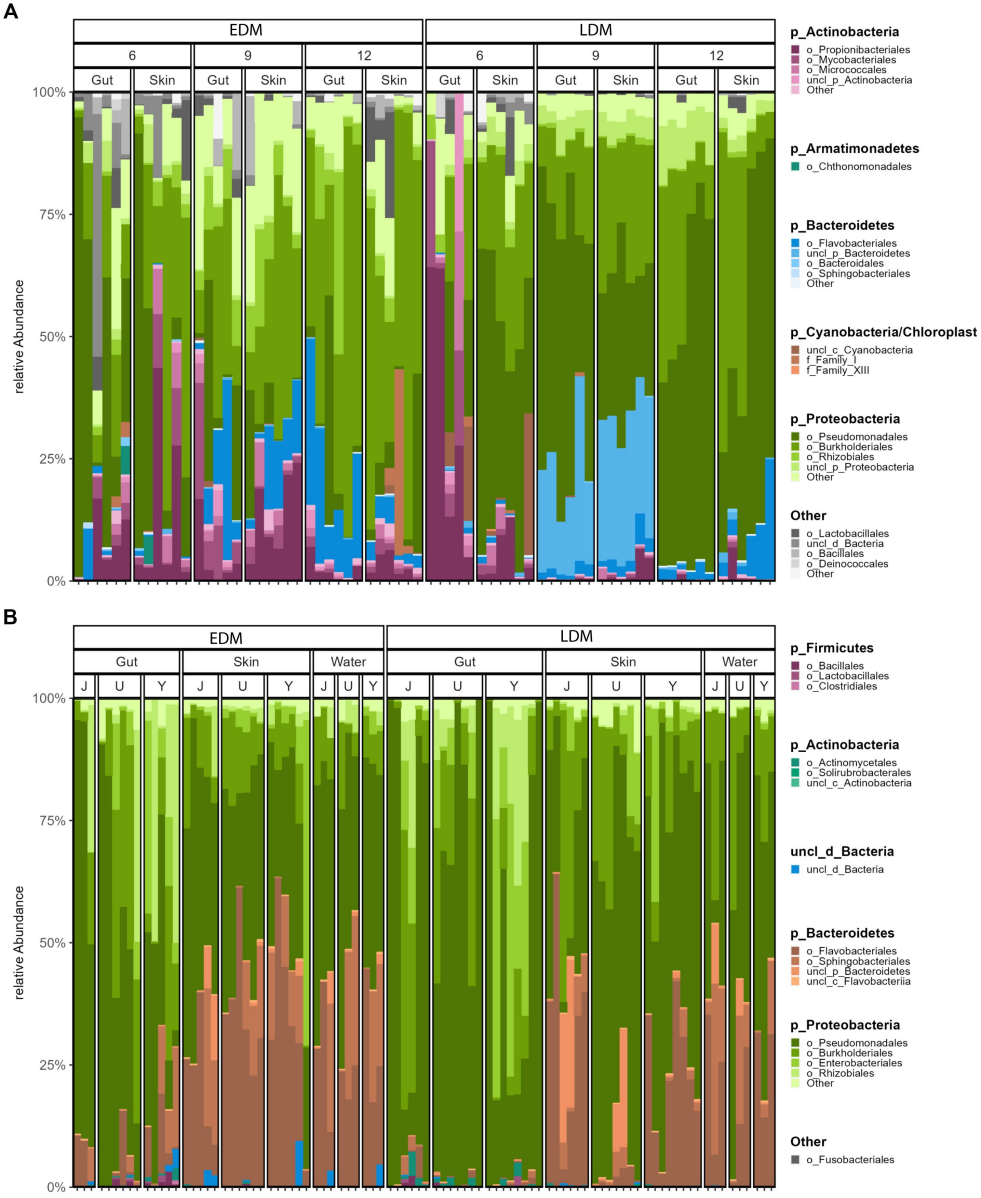
The relative bacterial community composition at the order level for all samples of Exp.1 **(A)** and Exp.2 **(B)**. For each phylum, the four most abundant orders are shown, others are summarized as “others.” ASVs not classified at order level are shown at the highest taxonomic level.

Pseudomonadales, Burkholderiales, Propionibacteriales, and Flavobacteriales were the dominant bacterial orders for all fish samples in both experiments (Figure 2). Interestingly, the order Pseudomonadales had a significantly higher relative abundance in the fish microbiota in LDM flasks, both in Exp.1 and Exp.2 (*t*-test, *p* < 0.001 and *p* = 0.013 for Exp.1 and Exp.2, respectively), and accounted on average for as much as 20–60% of the reads in the samples. Furthermore, Flavobacteriales was more abundant in the fish microbiota of EDM than the LDM samples in both experiments.

**Figure 3:**
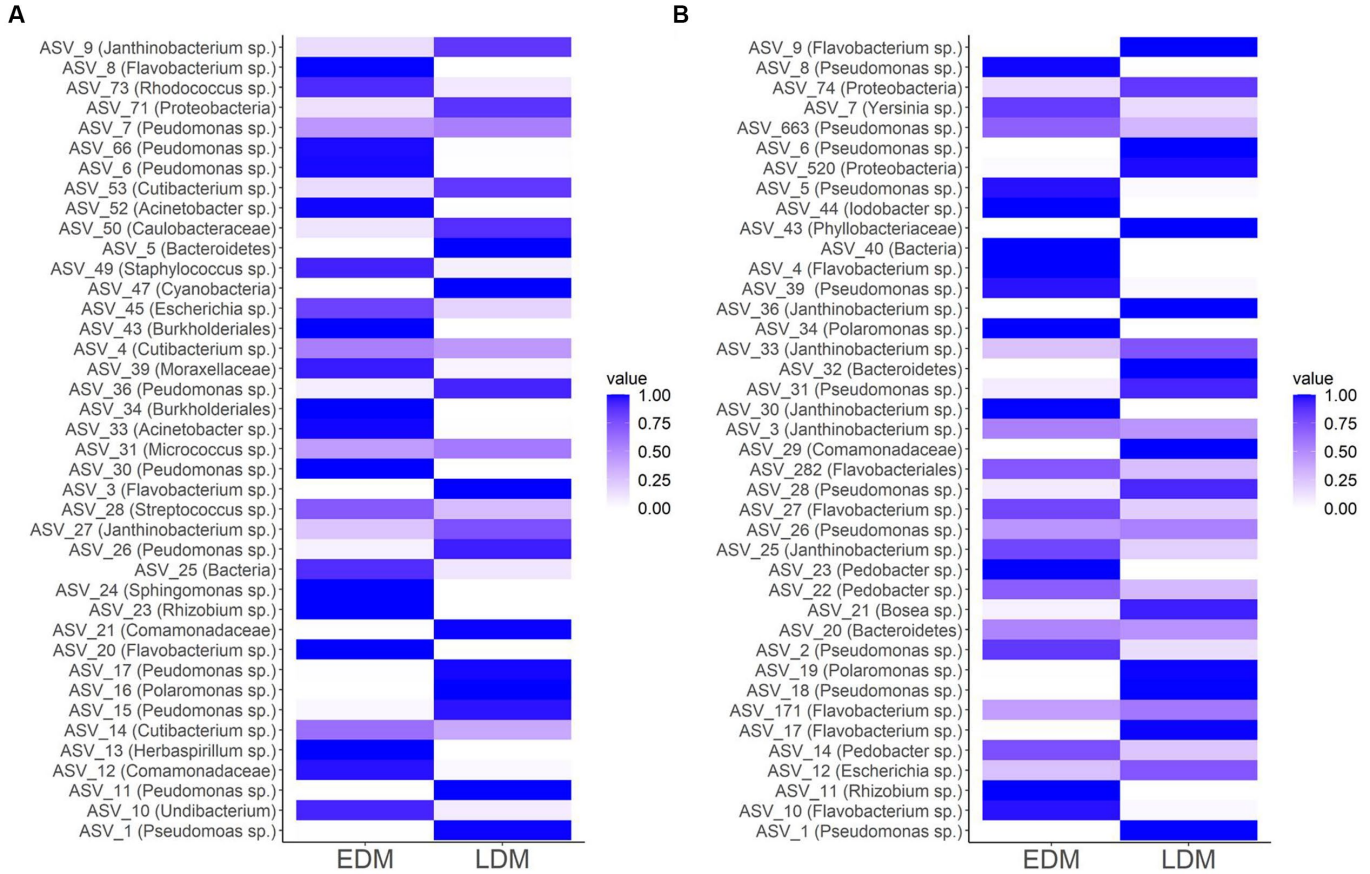
Heatmap showing the relative abundances of the Top 40 abundant ASVs in either EDM or LDM samples of Exp.1 **(A)** and Exp.2 **(B)**. Note that within this plot the ASVs are not sorted within their abundance but according to their number.

At ASV level, most of the abundant ASVs were exclusively present in either EDM or LDM samples (Figure 3 and Supplementary Figures S3, S4). Most samples, especially of the LDM group, were dominated by only a few ASVs that accounted for the majority of the reads (Supplementary Figures S3, S4). Accordingly, the evenness was significantly lower in LDM samples compared to EDM samples at 9 and 13 wph in Exp.1 (Mann–Whitney *U* test, *p* = 0.022 and 0.006 for 9 and 13 wph, respectively). There was however no significant difference in the evenness between EDM and LDM samples at 6 wph in Exp.1 and not in Exp.2.

**Figure 4:**
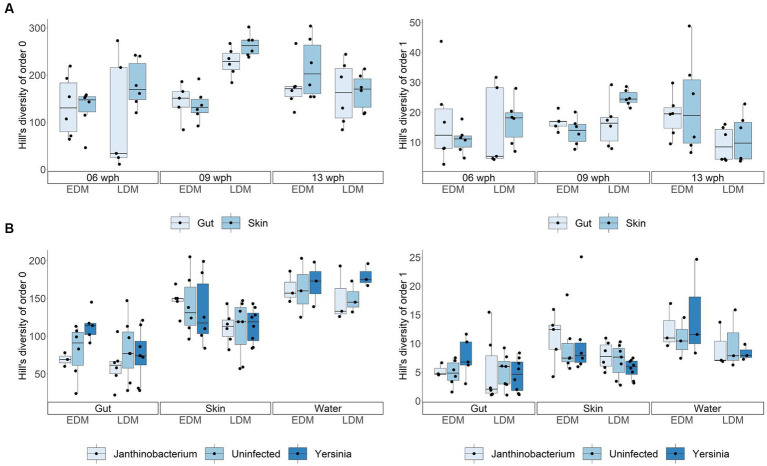
Hill’s diversity of order 0 (ASV richness) and order 1 (exponential Shannon index) for all samples from Exp.1 **(A)** and Exp.2 **(B)**. The box in the boxplots represent the median of all samples as well as the upper and lower quartiles. Whiskers include all samples except for outliers.

We examined the α-diversity by determining the ASV richness (Hill’s diversity of order 0) and Hill’s diversity of order 1. The α-diversity between EDM and LDM samples was similar in both gut and skin microbiota (Figure 4). There was no significant difference in Hill’s diversity of the order 0 (ASV richness) between the EDM and LDM microbiota in Exp.1 (Mann–Whitney *U* test, *p* > 0.05), except for samples taken at 9 wph (Mann–Whitney *U* test, *p* < 0.001), where LDM samples had a higher richness (Figure 4A). Further, the α-diversity measured as Hill’s diversity of order 1 was very similar between the EDM and LDM gut and skin microbiota (Mann–Whitney *U* test, *p* = 0.889; Figure 4A). In Exp.2, this was the case for both order 0 (Mann–Whitney *U* test, *p* = 0.610) and 1 (Mann–Whitney *U* test, *p* = 0.682; Figure 4B). These results show that even though the source microbiota strongly influenced the bacterial composition of the early Atlantic salmon gut and skin microbiota, it had little influence on the *α*-diversity.

### 3.3. Temporal development of the gut and skin microbiota throughout the yolk sac stage

We used the samples collected in Exp.1 to examine the temporal development of the skin and gut microbiota of the fish. A PCoA indicated that the microbiota was dynamic throughout the yolk sac stage, especially for samples from the LDM flasks (Figure 1A). A nested PERMANOVA test with flasks as sublevels showed that for both EDM and LDM samples (gut and skin samples analyzed together), the microbiota changed significantly both from 6 to 9 wph and from 9 to 13 wph (value of *p*s ≤ 0.001).

From 9 to 13 wph, the microbiota of the LDM flasks changed to a significantly larger extent than the EDM microbiota (Mann–Whitney *U* test, *p* < 0.001), as indicated by lower Bray–Curtis similarities between the samples (Supplementary Figure S1). Thus, the microbiota of fish that had been colonized by the lake water was less stable over time than that of the fish that had been colonized by their egg microbiota. This temporal development is reflected in the community composition at the order level: for the EDM samples, the relative abundance of Pseudomonadales decreased, while that of Flavobacteriales and Burkholderiales increased with increasing age (Figure 2A). For the LDM samples, Pseudomonadales remained the dominant order for most samples, even at the end of the yolk sac stage (Figure 2A). The microbiota of the fish also underwent major changes at the ASV level (Supplementary Figure S3B). This was particularly profound for the microbiota of the LDM samples. For example, even though the genus *Pseudomonas* was highly abundant at all sampling times for the LDM samples, different ASVs (classified as *Pseudomonas*) accounted for this high relative abundance at different age (e.g., ASV7 and 15 on 6 wph, ASV11, and 26 on 9 wph, and ASV1 and 17 on 13 wph; Supplementary Figure S3B). The ASV richness increased significantly for both the EDM and LDM samples over time (Kruskal–Wallis test, value of *p* = 0.019 and 0.001 for EDM and LDM, respectively), while Hill’s diversity of order 1 increased significantly only for the LDM samples (Kruskal–Wallis test, value of *p* = 0.169 and 0.008 for EDM and LDM, respectively; Figure 4A).

### 3.4. The effect of rearing flask on the larval microbiota

**Figure 5:**
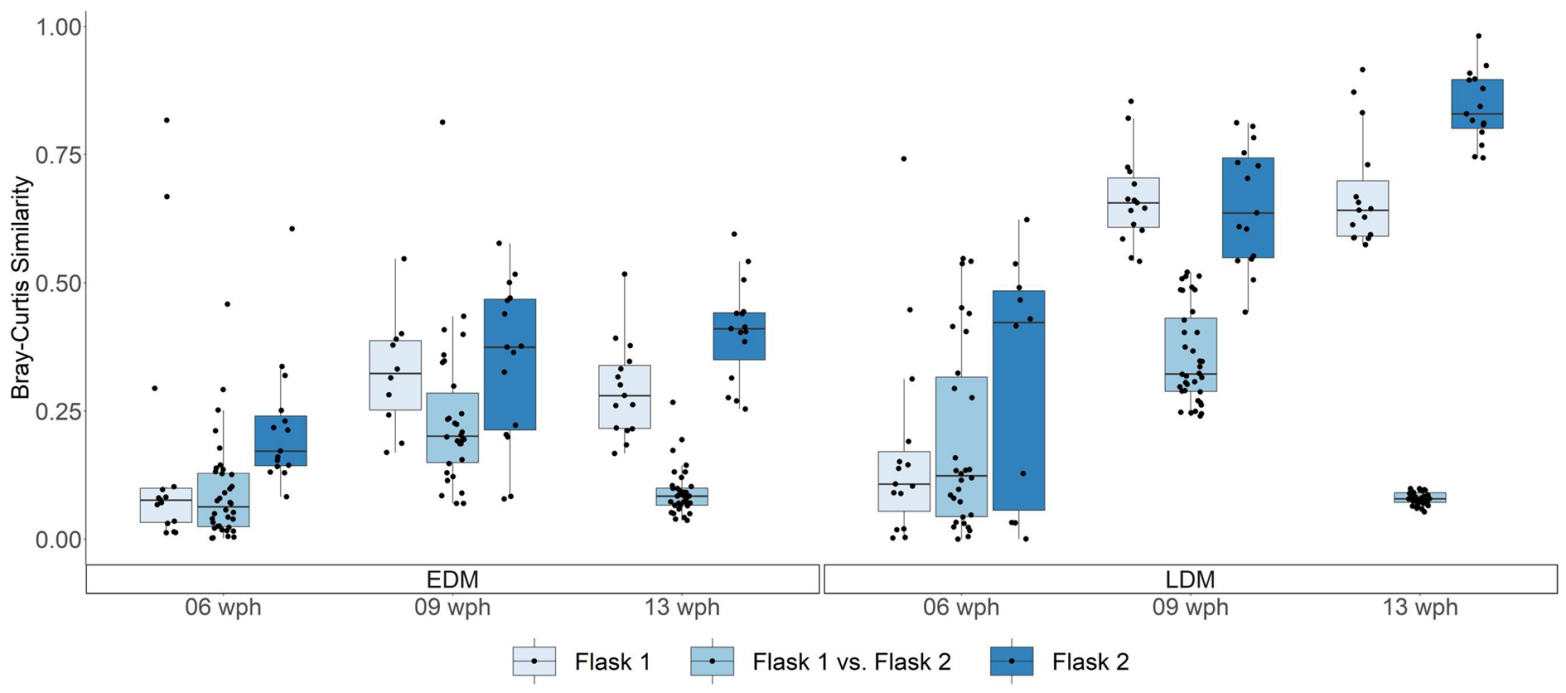
Boxplot showing Bray–Curtis similarities for comparisons of fish microbiota (both gut and skin) within and between replicate flasks for Exp.1. Each point represents a single comparison of two samples. The box in the boxplots represent the median of all samples as well as the upper and lower quartiles. Whiskers include all samples except for outliers.

The PCoA for the fish samples from Exp.1 (Figure 1A) indicated that the skin and gut microbiota differed between replicate rearing flasks. This was particularly clear for the LDM flasks at 13 wph. A PERMANOVA test revealed that the fish microbiota differed significantly between the two replicate flasks for each timepoint for both EDM and LDM samples (value of *p*s <0.05), except for LDM samples from 6 wph (*p* = 0.171). Interestingly, average Bray–Curtis similarities suggested that the fish microbiota both differed more between replicate LDM flasks and was more alike within replicate flasks (Figure 5). This was more pronounced at the last sampling time at 13 wph (Figure 5 and Supplementary Figure S5A). Also in Exp.2, the fish microbiota differed between replicate flasks, and again, this was especially profound for the LDM samples (Figure 1B and Supplementary Figure S5B). A PERMANOVA tests confirmed a significant difference in the fish microbiota between the three LDM replicate flasks (*p* ≤ 0.001), but not the EDM flasks (*p* = 0.109). Thus, the microbiota of fish colonized by their egg bacteria was more stable between replicate rearing flasks than that of fish colonized by the lake microbiota.

### 3.5. Comparison of skin, gut, and rearing water microbiota

For Exp.1, we collected skin and gut samples, but did not sample the rearing water. The PCoA (Figure 1A) and the community composition at the order level (Figure 2A) indicated that the microbiota of gut and skin samples were relatively similar and PERMANOVA tests did not show significant differences (*p*-values >0.05) between the gut and skin microbiota at any of the sampling times for neither the EDM nor the LDM samples. To avoid potential biases due to the effect of the replicate flask on the microbiota, we compared the Bray–Curtis similarities of gut and skin samples within replicate flasks. This indicated that the gut and skin microbiota differed for fish in the LDM flasks, and in the EDM flasks at 13 wph (Supplementary Figure S6). A Mann–Whitney *U* test showed that for the LDM flasks at 9 and 13 wph, the Bray–Curtis similarities were significantly lower for gut-skin comparisons than for skin-skin and gut-gut comparisons (*p* = 0.003 and 0.002 for 9 and 13 wph, respectively). The microbiota of gut and skin samples did not significantly differ in Hill’s diversity of order 0, 1 or evenness for any of the timepoints (Mann–Whitney *U* test, *p*-value >0.05; Figure 4A).

In Exp.2, we characterized the rearing water microbiota in addition to the gut and skin microbiota at 8 wph. Interestingly, the PCoA indicated that for the EDM rearing flasks, the skin and water microbiota seemed to be more alike to each other than to the gut microbiota (Figure 1B). For the LDM flasks, the samples clustered according to the replicate flask, and a potential higher similarity between skin and water samples was not obvious. Accordingly, a PERMANOVA test for the EDM samples showed that the gut microbiota differed significantly from both the skin and water (*p* = 0.013 and 0.003, respectively), whereas the microbiota of EDM skin and water samples did not differ significantly (PERMANOVA, *p* = 0.261). For the LDM samples, no significant difference was found between the microbiota of the different sample types (PERMANOVA, *p* > 0.05). However, the microbiota of both skin and water appeared to be characterized by a higher abundance of Flavobacteriales and Sphingobacteriales compared to the gut samples (Figure 2B). This might indicate differences in bacterial community compositions between gut and skin/water samples also for the LDM samples, even though this was not statistically significant in a PERMANOVA test. The *α*-diversity was highest for the water samples and lowest for the gut microbiota samples, both in terms of Hill’s diversity of order 0 and 1 (Figure 4B). The differences in α-diversity between the gut and skin samples were however only significant for the EDM samples (Kruskal–Wallis test, *p* = 0.020 and 0.038 for order 0 and 1, respectively) but not for LDM samples (Kruskal–Wallis test, *p* = 0.084 and 0.202 for order 0 and 1, respectively).

These results indicate that while the skin microbiota was similar to the water microbiota, a distinctive gut bacterial community was developing already in the yolk sac stage, prior to the onset of external feeding. Still, the differences between the gut and skin microbiota were small compared to the differences we observed in the fry microbiota between replicate flasks and between LDM and EDM samples (Figure 1).

### 3.6. Potential for manipulating the larval microbiota through exposure to bacterial isolates

In Exp.2, we further examined the potential for manipulating the microbiota of the fish by adding high concentrations of either the fish pathogen *Yersinia ruckeri* 06059 or the fish commensal *Janthinobacterium* sp. 3.108 to the rearing water of both EDM and LDM flask at 6 wph (2 weeks prior to bacterial sampling). By comparing the 16S rRNA gene sequences of these two strains (Supplementary Figure S7) with the ASV sequences, we identified ASV7 as *Y. ruckeri* 06059 and ASV3 and ASV15 (combined to ASV3-15; see Methods, “*Analysis of the Illumina sequencing data*”) as *J*. sp. 3.108.

Both strains successfully colonized the gut and skin of the salmon yolk sac fry (Supplementary Figure S8). As expected, ASV7 was generally not present in samples from flasks to which *Y. ruckeri* was not added and the relative abundance of ASV7 was significantly higher in samples taken from flasks to which *Y. ruckeri* 06059 was added (in both the EDM and LDM group; Mann–Whitney *U* test *p* = 0.012 and < 0.001, respectively). However, it varied strongly between individuals, from not observed for some samples and up to 50% in relative abundance for other samples, indicating that the colonization success for the *Y. ruckeri* isolate varied. Generally, the relative abundance of ASV7 was higher in gut samples than in skin or water samples (Supplementary Figure S8), however this was not significant (Kruskal–Wallis test *p* = 0.944 and *p* = 0.969 for EDM and LDM, respectively). Surprisingly, and in contrast to what we found for ASV7/*Y. ruckeri*, ASV3-15, representing *J*. sp. 3.108, was detected in considerable quantities in water, gut, and skin samples (on average around 5% in relative abundance), even for samples from flasks to which *J*. sp. 3.108 had not been added (Supplementary Figure S8). This was the case for samples from both EDM and LDM flasks, even though EDM and LDM samples were highly dissimilar in community composition at ASV level, with few highly abundant shared ASVs (Supplementary Figure S4). Furthermore, there was no significant difference in the relative abundance of ASV3-15 between samples from flasks that had been added *J. sp.* 3.108 and samples from flasks that had not been added this bacterial isolate (Mann–Whitney *U* test *p* = 0.305 and *p* = 0.682 for EDM and LDM, respectively). This means that the addition of *J*. sp. 3.108 to the fish flasks did not result in increased relative abundance of this strain in the gut and skin microbiota. Apart from ASV3, as many as 19 more ASVs were classified to the genus *Janthinobacterium* and these ASVs together had an average relative abundance of 10.3 ± 11.0% of all reads per sample. This, together with the fact that strain *J*. sp. 3.108 was originally isolated from the skin of salmon fry, might indicate a role of *Janthinobacterium* as a part of the commensal Atlantic salmon microbiota (see Discussion).

**Figure 6:**
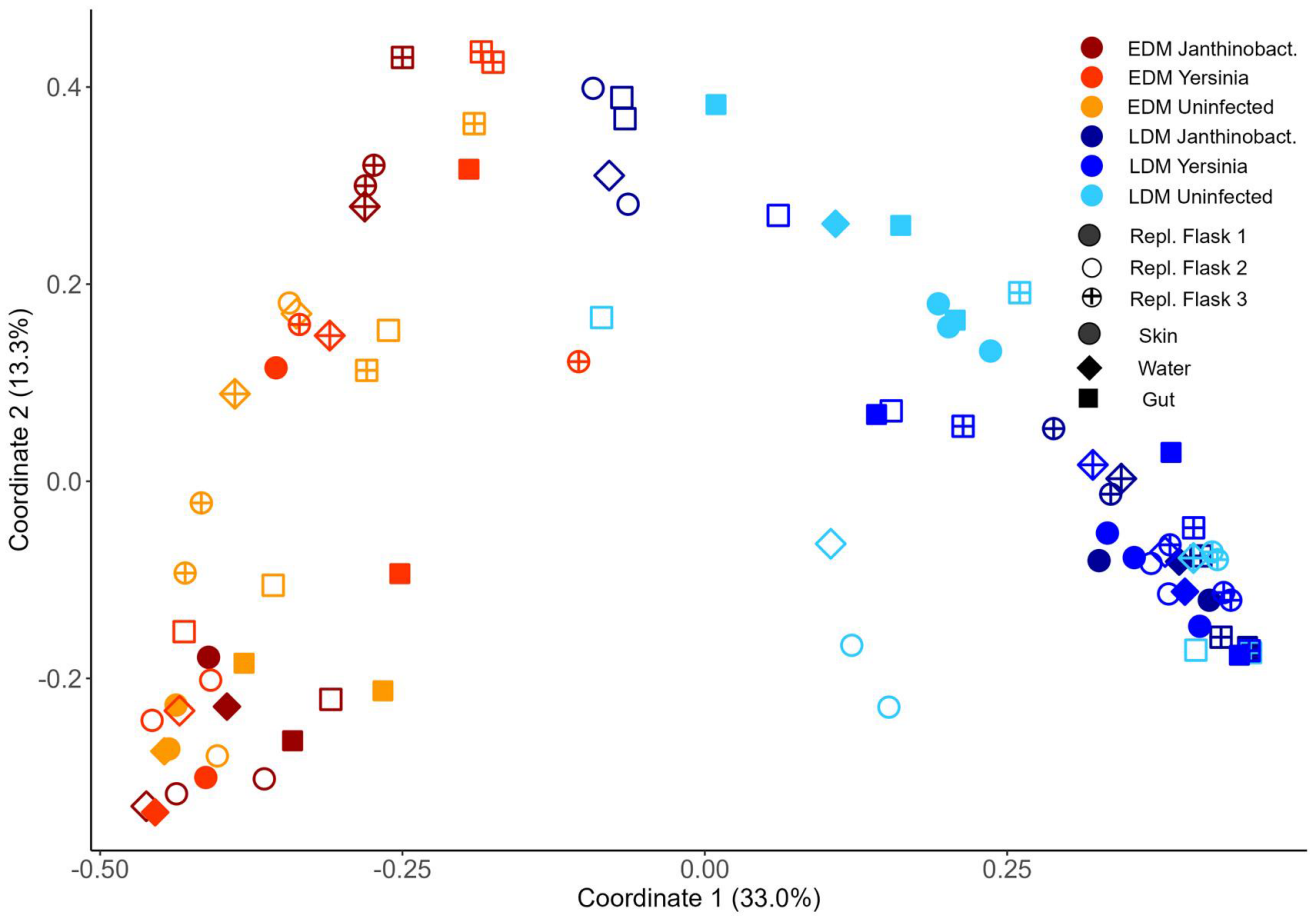
Ordination by PCoA based on the Bray–Curtis similarity of all samples from Exp.2, sampled 2  weeks after treatment.

A PCoA suggested that for EDM samples, there were no major differences in the fish’s microbiota between flasks that had been added *Y. ruckeri* or *J*. sp. 3.108 and flasks that had not been added bacterial isolates (Figure 6). A PERMANOVA confirmed that there was no significant difference in neither the gut nor the skin microbiota between EDM flasks added bacterial isolates and control flasks, not added bacterial isolates (*p* = 0.098 and *p* = 0.348, for gut and skin samples of the *Yersinia*-treatment and *p* = 0.468 and *p* = 0.225 for gut and skin samples of *Janthinobacterium*-treated samples, respectively). For the LDM samples however, the PCoA plot indicated that the fish’s microbiota was influenced by addition of *Y. ruckeri* and *J.* sp. 3.108 (Figure 6). A PERMANOVA test demonstrated that the skin microbiota, but not the gut microbiota, differed significantly between non-treated flasks and flasks that had been added bacterial isolates (*p* = 0.007 and 0.030 for *Y. ruckeri*-treated and *Janthinobacterium*-treated samples, respectively). However, a potential explanation for this observation could be the general difference in fish microbiota between replicate rearing flasks rather than the treatment with bacterial isolates *per se*. A nested PERMANOVA was performed to clarify this, however, due to the limited sample size, no conclusions could be drawn. Additionally, neither the richness, nor the exponential Shannon index were significantly affected by addition of *J.* sp. 3.108 or *Y. ruckeri* (Figure 4B).

Taken together, these findings suggest that neither addition of high loads of the fish pathogen *Y. ruckeri* nor of the presumed fish commensal *J*. sp. 3.108 to the rearing flasks lead to any major changes in the gut and skin microbiota of the Atlantic salmon yolk sac fry.

## 4. Discussion

The microbiota of fish is crucial for host health and development (Rawls et al., 2004), but little is known about the assembly of the fish microbiota just after hatching. In this study, we investigated the microbiota of the developing fish larvae that had been exposed to two different sources of microbiota present at hatching: either from the eggs of the fish (EDM; fish hatched under conventional, i.e., non-germ-free conditions) or from a freshwater lake (LDM; fish hatched under germ-free conditions and re-colonized). We found that the source microbiota had a strong influence on the skin and gut microbiota of the fish, as the microbiota differed significantly between the EDM and LDM group at all sampling timepoints. Interestingly, the microbiota of fish for which the source of bacteria was the egg (EDM) were more stable, both over time and between replicate rearing flasks, than fish colonized by lake water bacteria (LDM). A possible reason for this might be that egg-derived microbes were better adapted to colonizing the fish, whereas the bacterial populations in the lake water-microbiota were probably poorly adapted for colonization of the fish. This may have increased the significance of stochastic processes, such as ecological drift, that play an important role in the initial community assembly (Dini-Andreote et al., 2015; Gu et al., 2021). Vestrum et al. (2020) showed that drift was important for creating variation in the microbiota between individuals in rearing systems with Atlantic cod larvae. Thus, a stronger influence of drift on the community assembly could explain the divergence in the fish microbiota between replicate rearing flasks.

Previous studies of fish in larger rearing systems, for example for Atlantic cod larvae (Bakke et al., 2013) and Atlantic salmon (Schmidt et al., 2016; Minich et al., 2020), have also demonstrated that the fish microbiota differed between replicate rearing tanks. Interestingly, we observed that the effect was more prominent when comparisons were based on the abundance-based Bray–Curtis similarity than on the presence/absence-based Sørensen-Dice similarity (Supplementary Figure S9). This indicates that the effect arose rather due to differences in the relative abundances of ASVs than the presence of distinct ASVs. In Exp.2, we found that the water and fish microbiota was similar within each rearing flask, and that a distinctive system microbiota developed in each replicate rearing flasks, although the same lake water was used as source community, and that the same bacterial populations thus were present during the initial bacterial colonization of the fish. This indicates that the water microbiota has a stronger influence on the fish microbiota than the selection pressure in the gut and skin of the fry. Comparisons of water and fish microbiota throughout the yolk sac stage might bring new insight about the interrelationship between the water and fish microbiota during the establishment and development of the early fish microbiota. However, unfortunately, we did not characterize the rearing water microbiota in Exp.1.

The finding that the source of bacteria present in the environment after hatching had a major impact on the composition and the stability of the fry’s microbiota, points to the possibility for steering the microbiota of the yolk sac fry by manipulating the microbial environments upon hatching. This might have an applied potential in the aquaculture industry, where eggs are routinly disinfected prior to the distribution to hatcheries. In principal, this could be a strategy to counteract negative host – microbe interactions and to develop robust fry by, e.g., introducing probiotic strains. However, research is needed to identify strategies for obtaining this, and to investigate the consequences in terms of host responses.

The differences in the larval microbiota between the EDM and LDM flasks were more profound when PCoA was based on Bray–Curtis similarities than on the weighted UniFrac similarity, which also takes into account the phylogenetic distances between the ASVs. This indicates that the fish in EDM and LDM flasks were colonized by different bacterial populations, which represent related taxa, and thus, that certain phylogenetic groups were selected for on the mucosal surfaces of the fish. Proteobacteria, Bacteroidetes and Actinobacteria were the most abundant phyla of the yolk sac fry microbiota. These phyla were also found to be highly abundant in the microbiota of Atlantic salmon yolk sac fry in a study by Lokesh et al. (2019). They characterized the egg microbiota and followed the Atlantic salmon gut microbiota until the fish were fully developed but included only one sample between hatching and onset of active feeding. Both our and their study found that Proteobacteria was the dominating phylum in the yolk sac fry, and we further found that Actinobacteria were strongly present in the early timepoints and later decreased. These findings are in line with the conclusion of Borges et al. (2021), that summarized for different fish species that Proteobacteria, Bacteroidetes, Firmicutes and Actinomycetes are the dominant phyla in the fish larvae gut (Borges et al., 2021). The main bacterial phyla of the skin microbiota in juvenile and adult Atlantic salmon have been found to be Proteobacteria, Bacteroidetes and Firmicutes (Lokesh and Kiron, 2016; Minniti et al., 2017; Wynne et al., 2020; Bugten et al., 2022) and our study shows that they were highly abundant already at the larval stage.

In Exp.1, we observed that the gut and skin microbiota underwent major changes throughout the yolk sac stage. This was particularly profound at the ASV level and for LDM samples, and very few ASVs were highly abundant at all sampling timepoints. Interestingly, even though Pseudomonadales dominated the fish microbiota in LDM flasks at all sampling times, this phylum was represented by distinct ASVs at the three sampling times, indicating that distinct *Pseudomonas* populations colonized the fish at different ages. Also in other vertebrates and fish species, it has been observed that the early microbiome is dynamic, and is only stabilizing later in life (Schloss et al., 2012; Chen and Garud, 2022; Woodruff et al., 2022; Xiao et al., 2022). We further observed large interindividual variation in the microbiota, an observation often made in other aquatic larvae (e.g., Verschuere et al., 1999; Stephens et al., 2016; Vestrum et al., 2020), which has been suggested to be a consequence of ecological drift (Vestrum et al., 2020).

For adult fish, several studies have shown that the skin and gut of fish harbors distinct microbial communities (e.g., Lowrey et al., 2015; Sylvain et al., 2020). However, few studies have focused on the skin microbiota of fish larvae (e.g., Dodd et al., 2020), and little is known about the diverging development of the gut and skin microbiota in the early developmental stages, especially prior to onset of active feeding. Already at 7 dph, long before the fish starts to feed, the anus and the mouth of Atlantic salmon is opened and therefore available for bacterial colonization (Sahlmann et al., 2015). Here, we dissected out the fish guts and studied the development of both the gut and skin microbiota prior to the onset of external feeding. Both in Exp.1 and 2 we found indications that the skin and gut microbiota started to differentiate already at 8–9 wph, several weeks before the yolk sac was consumed. We observed however, that the gut was filled with yolk material (not shown), which might provide nutrients to the gut microbiota. Sahlmann et al. (2015) further observed that distinct gut organs and structures formed already from 7 dph on. This structuring of the gut might already provide colonization space for bacterial populations filling different niches, resulting in a distinct gut microbiota.

Moreover, Exp.2 showed that the microbial skin communities resembled the water microbiota, whereas the gut microbiota differed from the water and skin microbiota. In studies of Gilthead Sea Bream and Atlantic cod larvae the microbiota of the whole fish (no differentiation between gut and skin) was found to differ from the water microbiota (Bakke et al., 2015; Nikouli et al., 2019; Vestrum et al., 2020), indicating that the selection of bacterial populations differed between the water and mucosal surfaces of the larvae. In our study we could now show that in the larval stage it indeed appears to be only the gut microbiota that is different from the water microbiota, not the skin microbiota. Studies in adult fish report that the skin microbiota is distinctive and differs from the surrounding water microbiota (Razak et al., 2019; Gomez and Primm, 2021). It would be interesting to further investigate to which extent the skin and water microbiota diverge throughout the yolk sac stage.

In Exp.2, we further investigated the potential of manipulating the fish’s microbiota by addition of one of two bacterial isolates in high densities (a theoretical final concentration of 10^7^ CFUml^−1^) 6 weeks after the fish had hatched. Either the fish pathogen *Y. ruckeri* or the presumed non-pathogenic fish commensal *Janthinobacterium* sp. 3.108 was added to EDM and LDM flasks. Surprisingly, the ASV corresponding to the *J*. sp. 3.108 isolate (or a strain with the same partial 16S rDNA gene sequence) was found in the microbiota of fish from all flasks, also those that had not been added the isolate. The relative abundances of that ASV varied extensively between individuals, but the average relative abundances did not increase in samples from flasks that had been added *J*. sp. 3.108. Accordingly, we found that addition of the commensal *J*. sp. 3.108 did not significantly change the microbiota. The fish pathogen *Y. ruckeri* was not present in significant amounts in flasks to which we did not add it. In flasks to which we added it, its abundance among individuals was highly variable and mainly present in low relative abundances. It further did not have a large impact on the skin and gut microbiota. These results may indicate that the microbiota of the larval Atlantic salmon is resistant to invasion by introduced bacterial strains. Skjermo et al. (2015) showed that none of the four probiotic candidate bacterial strains originally isolated from cod larvae were able to establish themselves as part of the microbiota of Atlantic cod larvae. Further, Puvanendran et al. (2021) found that their probiotic *Carnobacterium* isolate could not establish itself in Atlantic cod larvae. This shows that manipulating the microbiota of fish with, e.g., probiotic strains might be difficult to achieve already in the larval stage, when the fish’s microbiota is still unstable. As discussed above, manipulating the microbial environments at hatching might be a better strategy for influencing the early fish microbiota.

Apart from the *Janthinobacterium* strain we added (strain 3.108), we also found several other ASVs classified as “*Janthinobacterium*” in high relative abundances. Strain *J*. sp. 3.108 was originally isolated from the skin of Atlantic salmon fry from a commercial RAS, and its 16S rRNA gene sequence is highly similar to the *Janthinobacterium lividum* type strain (99% similarity over the whole 16S rRNA gene, data not shown). *J. lividum* commonly occurs in freshwater (Pantanella et al., 2007) and is a commensal of both the amphibian (Brucker et al., 2008; Becker et al., 2009) and human (Grice et al., 2008; Ramsey et al., 2015) skin microbiota, and it has antagonistic properties against fungi and bacteria (Munakata et al., 2021). *Janthinobacterium* spp. have also been found in tank biofilms of fish farms for rainbow trout (Nakamura et al., 2002; Testerman et al., 2021). A *J. lividum* strain was shown to be capnophilic (Valdes et al., 2015), meaning it thrives under high concentrations of CO_2_. The salmon larvae exchange gas mainly through the skin, and this could be an explanation for the presence of *Janthinobacterium* in the skin microbiota. As we also found high abundances of several ASVs classified as *Janthinobacterium* associated with the skin and gut samples in both experiments, we propose that strains from the genus *Janthinobacterium* are commensal bacteria for Atlantic salmon larvae. Members of *Janthinobacterium* have also been found in the intestine of adult Atlantic salmon (Wang et al., 2018).

In contrast to *Janthinobacterium*, *Y. ruckeri* is a well-known pathogen in later life stages of the salmon (e.g., Kumar et al., 2015). However, it appears as if no lethal disease was triggered by the addition of *Y. ruckeri*, even though the strain used in this study (*Y. ruckeri* 06059) has successfully been used to inflict mortality in Atlantic salmon fry (Haig et al., 2011). A possible reason for this might be that either the temperature used here was too low (14°C) or that the yolk sac fry was not developed enough for *Y. ruckeri* to induce mortality.

In this study, RNA extracts were available for characterization of the fish microbiota for the samples collected in Exp.1, while in Exp.2, the analyses of the microbiota were based on DNA. RNA-based microbiota analyses are assumed to reflect the actively growing populations in the microbial communities to a larger extent as compared to DNA-based analyses, which will also represent inactive bacterial cells. As these two experiments also differed in other parts of the methodology (e.g., in how the nucleic acids were extracted), it is not possible to compare these two datasets directly. We therefore analyzed the data from the two experiments separately and compared how they answered our research questions. We found that the key findings were shared for the two experiments, as for both experiments the fish microbiota varied between LDM and EDM flask and also between replicate rearing flasks. We further saw differences between gut and skin samples in both the RNA-based and DNA-based data. Therefore, even though different approaches were used in the two experiments, both answered our research questions in similar ways, which indicates that our findings are robust.

In conclusion, we showed that the skin and gut microbiota were similar, but started diverging during the yolk sac stage, several weeks before the yolk sac was consumed. The skin microbiota was more similar to the water microbiota than the gut microbiota. Furthermore, the microbiota differed profoundly between fish that had been conventionally reared, i.e., the egg microbiota was the only source of bacteria (EDM), and fish that had been made germ-free and were then colonized by using lake water as a source for bacteria (LDM). Proteobacteria, Bacteroidetes and Actinobacteria were the most abundant phyla in the fry microbiota. Both the skin and gut microbiota were highly dynamic and underwent major changes at the ASV level throughout the yolk sac stage, and this was particularly evident for fry reared in LDM flasks. The fry microbiota differed profoundly between replicate rearing flasks, and again, this was particularly evident for the LDM flasks. Thus, the fry reared in EDM flasks had a more stable microbiota, both between rearing flasks and over time. Additions of high doses of the pathogen *Y. ruckeri* to fish flasks did not cause mortality. Addition of *Y. ruckeri* had only minor impact on the community composition. Finally, we exposed the fry to high doses of a *Janthinobacterium* sp. isolate and found no effects on the fry microbiota. An ASV sequence corresponding to the one for the added *J*. sp. isolate was abundant in most fry samples and indicated that this represented a commensal member of the early fry microbiota.

## Data availability statement

The datasets presented in this study can be found in online repositories. The names of the repository/repositories and accession number(s) can be found at: https://www.ebi.ac.uk/ena, ERS14440101–ERS14440192.

## Ethics statement

Ethical review and approval was not required for the animal study because the Atlantic salmon yolk sac fry are not defined as living animals according to Norwegian legislation, therefore no ethical review was required.

## Author contributions

MD and AF conducted the fish experiments. AF and EL prepared the 16S libraries and analyzed the resulting data. AF, OV, and IB conceived and designed the experiments. AF wrote the first draft of the manuscript. All authors contributed to the article and approved the submitted version.

## Funding

This work was supported by the Department of Biotechnology and Food Science at NTNU, and by the Research Council of Norway (RCN) through their funding of the FRIPRO project number 262929.

## Conflict of interest

The authors declare that the research was conducted in the absence of any commercial or financial relationships that could be construed as a potential conflict of interest.

## Publisher’s note

All claims expressed in this article are solely those of the authors and do not necessarily represent those of their affiliated organizations, or those of the publisher, the editors and the reviewers. Any product that may be evaluated in this article, or claim that may be made by its manufacturer, is not guaranteed or endorsed by the publisher.

## Footnotes

^1^ https://cran.rstudio.com/

## Supplementary material

The Supplementary material for this article can be found online at:

https://www.frontiersin.org/articles/10.3389/fmicb.2023.1177972 /full#supplementary-material
