# Supplementary material for: The stability and composition of the gut and skin microbiota of Atlantic salmon throughout the yolk sac stage
Source: Front Microbiol. 2023 Jul 6;14:1177972. doi: 10.3389/fmicb.2023.1177972 (PMC10358989; doi:10.3389/fmicb.2023.1177972)
Supplement: Supplementary file 1 [file Data_Sheet_1.PDF]

Supplementary Material

The stability and composition of the gut and skin microbiota of Atlantic salmon throughout the yolk sac stage

Alexander W. Fiedler[1], Martha K. R. Drågen[1], Eirik D. Lorentsen[1], Olav Vadstein[1], Ingrid Bakke[*1]

[1]Department of Biotechnology and Food Science, Norwegian University of Science and Technology, Trondheim, Norway

*** Correspondence:**
Ingrid Bakke
Ingrid.bakke@ntnu.no

Supp. Table 1: α-diversity for all samples from Exp.1. The columns D0-Inf. represent the α-diversity as Hill's diversity of the order 0 - infinite. E1 represents the evenness. The sample coding is as follows: 6/9/13: samples from 6 wph, 9 wph or 13 wph; E/L: EDM or LDM samples; 1-6: Replicate fish; G/S: Sample type i.e. gut or skin. Sequencing coverage was calculated by dividing the richness (D0) by the Chao1 index.

Sample	Treatment	Sample Type	wph	Flask	D0	D1	D2	Inf.	E1	Chao1	Seq. coverage
6E1G	EDM	Gut	6	1	107	2.67	1.65	1.31	0.025	112	0.96
6E2G	EDM	Gut	6	1	64	8.04	3.74	2.03	0.126	132	0.49
6E3G	EDM	Gut	6	1	71	7.92	3.70	2.01	0.112	84	0.84
6E4G	EDM	Gut	6	2	219	16.70	4.38	2.14	0.076	291	0.75
6E5G	EDM	Gut	6	2	193	43.71	24.02	8.76	0.226	197	0.98
6E6G	EDM	Gut	6	2	154	22.64	11.51	4.54	0.147	175	0.88
6E1S	EDM	Skin	6	1	115	4.74	2.26	1.54	0.041	155	0.74
6E2S	EDM	Skin	6	1	158	11.51	4.84	2.42	0.073	309	0.51
6E3S	EDM	Skin	6	1	46	7.61	4.63	2.51	0.165	60	0.77
6E4S	EDM	Skin	6	2	152	12.36	4.02	2.06	0.081	177	0.86
6E5S	EDM	Skin	6	2	143	17.79	9.76	5.02	0.124	156	0.92
6E6S	EDM	Skin	6	2	155	10.76	4.24	2.26	0.069	169	0.92
9E1G	EDM	Gut	9	1	132	21.37	14.34	7.78	0.162	161	0.82
9E2G	EDM	Gut	9	1	186	17.06	6.88	2.91	0.092	203	0.92
9E4G	EDM	Gut	9	2	84	13.29	6.22	2.77	0.158	116	0.73
9E5G	EDM	Gut	9	2	165	16.96	7.23	3.35	0.103	190	0.87

9E6G	EDM	Gut	9	2	151	15.42	8.67	4.70	0.102	171	0.88
9E1S	EDM	Skin	9	1	153	16.22	8.75	4.43	0.106	166	0.92
9E2S	EDM	Skin	9	1	118	12.72	8.03	5.14	0.108	137	0.86
9E3S	EDM	Skin	9	1	192	20.09	10.79	4.66	0.105	239	0.80
9E4S	EDM	Skin	9	2	92	7.67	3.58	2.00	0.083	101	0.91
9E5S	EDM	Skin	9	2	127	9.52	4.85	2.70	0.075	148	0.86
9E6S	EDM	Skin	9	2	136	15.25	7.78	4.41	0.112	141	0.97
13E1G	EDM	Gut	12	1	150	19.60	11.44	5.58	0.131	189	0.79
13E2G	EDM	Gut	12	1	175	22.22	12.49	6.88	0.127	186	0.94
13E3G	EDM	Gut	12	1	267	29.81	11.41	4.37	0.112	300	0.89
13E4G	EDM	Gut	12	2	176	13.16	5.42	2.72	0.075	197	0.89
13E5G	EDM	Gut	12	2	167	19.39	9.33	3.97	0.116	199	0.84
13E6G	EDM	Gut	12	2	121	9.49	4.36	2.36	0.078	173	0.70
13E1S	EDM	Skin	12	1	276	26.22	8.42	3.39	0.095	300	0.92
13E2S	EDM	Skin	12	1	226	32.41	13.79	5.51	0.143	269	0.84
13E3S	EDM	Skin	12	1	304	48.82	24.19	8.30	0.161	408	0.75
13E4S	EDM	Skin	12	2	179	9.04	4.09	2.51	0.050	197	0.91
13E5S	EDM	Skin	12	2	154	6.58	2.26	1.51	0.043	195	0.79
13E6S	EDM	Skin	12	2	154	11.77	4.78	2.93	0.076	174	0.89
6L1G	LDM	Gut	6	1	11	4.28	3.70	3.08	0.389	12	0.92
6L2G	LDM	Gut	6	1	34	5.32	3.98	3.10	0.156	35	0.99
6L3G	LDM	Gut	6	1	216	31.67	17.02	5.93	0.147	312	0.69
6L5G	LDM	Gut	6	2	25	4.61	4.12	3.53	0.184	28	0.89

6L6G	LDM	Gut	6	2	273	28.23	10.87	5.32	0.103	343	0.80
6L1S	LDM	Skin	6	1	120	6.97	2.70	1.67	0.058	140	0.86
6L2S	LDM	Skin	6	1	144	9.63	3.49	1.93	0.067	161	0.90
6L3S	LDM	Skin	6	1	242	28.01	10.88	4.74	0.116	255	0.95
6L4S	LDM	Skin	6	2	161	17.96	10.15	5.12	0.112	220	0.73
6L5S	LDM	Skin	6	2	178	18.36	6.60	2.91	0.103	214	0.83
6L6S	LDM	Skin	6	2	240	20.37	7.68	4.04	0.085	292	0.82
9L1G	LDM	Gut	9	1	223	7.89	3.05	1.87	0.035	264	0.85
9L2G	LDM	Gut	9	1	184	8.78	3.61	2.12	0.048	229	0.80
9L3G	LDM	Gut	9	1	235	18.59	6.61	2.83	0.079	269	0.87
9L4G	LDM	Gut	9	2	208	15.40	7.68	3.48	0.074	274	0.76
9L5G	LDM	Gut	9	2	250	17.41	5.82	2.65	0.070	291	0.86
9L6G	LDM	Gut	9	2	267	29.22	12.86	6.00	0.109	350	0.76
9L1S	LDM	Skin	9	1	274	24.64	8.81	3.48	0.090	327	0.84
9L2S	LDM	Skin	9	1	302	24.23	7.99	3.41	0.080	350	0.86
9L3S	LDM	Skin	9	1	243	21.44	8.36	4.25	0.088	282	0.86
9L4S	LDM	Skin	9	2	251	23.03	7.91	3.24	0.092	286	0.88
9L5S	LDM	Skin	9	2	238	28.59	9.54	3.64	0.120	250	0.95
9L6S	LDM	Skin	9	2	274	27.23	8.97	3.47	0.099	352	0.78
13L1G	LDM	Gut	12	1	220	14.90	5.79	2.92	0.068	273	0.81
13L2G	LDM	Gut	12	1	196	12.57	4.88	2.56	0.064	230	0.85
13L3G	LDM	Gut	12	1	244	16.01	5.28	2.61	0.066	287	0.85

13L4G	LDM	Gut	12	2	102	4.34	2.08	1.46	0.043	127	0.80
13L5G	LDM	Gut	12	2	130	3.99	1.82	1.36	0.031	153	0.85
13L6G	LDM	Gut	12	2	84	4.44	2.14	1.48	0.053	102	0.82
13L1S	LDM	Skin	12	1	174	14.83	5.65	2.71	0.085	203	0.86
13L2S	LDM	Skin	12	1	198	22.80	9.57	4.16	0.115	229	0.86
13L3S	LDM	Skin	12	1	213	17.29	6.54	3.13	0.081	244	0.87
13L4S	LDM	Skin	12	2	167	4.35	1.83	1.36	0.026	250	0.67
13L5S	LDM	Skin	12	2	120	3.72	1.80	1.35	0.031	145	0.83
13L6S	LDM	Skin	12	2	118	4.67	2.34	1.58	0.040	138	0.86

Supp. Table 2: α-diversity indices for all samples of Exp.2. The columns D0 - Inf. represent the α-diversity as Hill's diversity of the order 0 - infinite. E1 represents the evenness. Sample name's coding is as follows: E/L: EDM or LDM samples; J/U/Y: Samples treated with *Janthinobacterium*, Untreated or *Yersinia*; 1/2/3: Replicate flask 1, 2 or 3; G/S/W: Sample type i.e. gut, skin or water; 1/2/3: Replicate fish no. 1, 2 or 3. Sequencing coverage was calculated by dividing the richness (D0) by the Chao1 index.

Sample	Bact. Status	Treatment	Sample Type	Rep. Flask	D0	D1	D2	Inf.	E1	Chao1	Seq. coverage
EJ1-G2	EDM	Janthinobacterium	Gut	J1	60	4.72	3.58	2.40	0.079	67	0.89
EJ1-S3	EDM	Janthinobacterium	Skin	J1	146	13.18	6.16	2.96	0.090	162	0.90
EJ1-W	EDM	Janthinobacterium	Water	J1	146	10.97	4.58	2.37	0.075	199	0.74
EJ2-G3	EDM	Janthinobacterium	Gut	J1	69	4.53	3.12	2.52	0.066	81	0.85
EJ2-S2	EDM	Janthinobacterium	Skin	J2	120	4.21	2.19	1.53	0.035	148	0.81
EJ2-S3	EDM	Janthinobacterium	Skin	J2	150	8.99	5.02	2.88	0.060	202	0.74
EJ2-W	EDM	Janthinobacterium	Water	J2	157	9.61	6.06	4.45	0.061	182	0.86
EJ3-G2	EDM	Janthinobacterium	Gut	J3	78	6.65	3.82	2.77	0.085	80	0.98
EJ3-S2	EDM	Janthinobacterium	Skin	J3	150	15.93	8.25	3.80	0.106	160	0.91
EJ3-S3	EDM	Janthinobacterium	Skin	J3	169	12.44	6.89	3.36	0.074	227	0.74
EJ3-W	EDM	Janthinobacterium	Water	J3	186	16.98	10.42	5.60	0.091	207	0.90
EU1-G2	EDM	Untreated	Gut	U1	24	1.54	1.24	1.12	0.064	27	0.89
EU1-G3	EDM	Untreated	Gut	U1	54	3.33	2.36	1.78	0.062	85	0.63
EU1-S2	EDM	Untreated	Skin	U1	96	5.67	3.68	2.70	0.059	143	0.67
EU1-S3	EDM	Untreated	Skin	U1	138	6.60	3.69	2.41	0.048	189	0.73
EU1-W	EDM	Untreated	Water	U1	160	10.43	5.43	3.99	0.065	204	0.78

EU2-G2	EDM	Untreated	Gut	U2	99	7.50	3.49	2.03	0.076	113	0.88
EU2-G3	EDM	Untreated	Gut	U2	113	5.41	3.78	2.52	0.048	133	0.85
EU2-S2	EDM	Untreated	Skin	U2	124	7.51	4.61	2.86	0.061	179	0.69
EU2-S3	EDM	Untreated	Skin	U2	111	7.41	4.21	2.93	0.067	174	0.64
EU2-W	EDM	Untreated	Water	U2	125	7.42	4.55	2.57	0.059	148	0.84
EU3-G2	EDM	Untreated	Gut	U3	83	4.28	2.17	1.51	0.052	98	0.85
EU3-G3	EDM	Untreated	Gut	U3	107	7.02	3.00	1.79	0.066	116	0.92
EU3-S2	EDM	Untreated	Skin	U3	174	10.84	5.20	2.77	0.062	185	0.94
EU3-S3	EDM	Untreated	Skin	U3	205	18.47	9.18	3.95	0.090	221	0.93
EU3-W	EDM	Untreated	Water	U3	203	14.52	8.89	5.10	0.072	236	0.86
EY1-G2	EDM	Yersinia	Gut	Y1	102	6.23	3.62	2.73	0.061	126	0.81
EY1-G3	EDM	Yersinia	Gut	Y1	91	2.98	2.29	2.12	0.033	131	0.70
EY1-S2	EDM	Yersinia	Skin	Y1	125	8.31	4.50	2.71	0.066	145	0.86
EY1-S3	EDM	Yersinia	Skin	Y1	110	6.45	4.26	2.79	0.059	153	0.72
EY1-W	EDM	Yersinia	Water	Y1	139	8.34	5.39	3.75	0.060	157	0.88
EY2-G2	EDM	Yersinia	Gut	Y2	145	10.28	6.74	4.44	0.071	173	0.84
EY2-S2	EDM	Yersinia	Skin	Y2	101	7.45	4.26	2.34	0.074	107	0.95
EY2-S3	EDM	Yersinia	Skin	Y2	184	10.68	5.98	3.36	0.058	222	0.83
EY2-W	EDM	Yersinia	Water	Y2	173	11.57	6.80	4.28	0.067	191	0.91
EY3-G2	EDM	Yersinia	Gut	Y3	114	11.63	6.57	3.26	0.102	134	0.85
EY3-G3	EDM	Yersinia	Gut	Y3	117	6.78	3.10	1.84	0.058	132	0.89
EY3-S2	EDM	Yersinia	Skin	Y3	199	25.10	13.31	6.01	0.126	227	0.88
EY3-S3	EDM	Yersinia	Skin	Y3	84	5.95	3.08	1.86	0.071	97	0.86

EY3-W	EDM	Yersinia	Water	Y3	198	24.66	14.31	6.47	0.125	241	0.82
LJ1-G1	LDM	Janthinobacterium	Gut	J1	22	1.04	1.01	1.00	0.047	36	0.61
LJ1-G2	LDM	Janthinobacterium	Gut	J1	48	1.18	1.05	1.02	0.025	51	0.95
LJ1-S1	LDM	Janthinobacterium	Skin	J1	96	5.83	3.14	1.99	0.061	106	0.90
LJ1-S2	LDM	Janthinobacterium	Skin	J1	82	4.93	3.40	2.39	0.060	84	0.98
LJ1-W	LDM	Janthinobacterium	Water	J1	126	6.87	3.33	2.15	0.055	168	0.75
LJ2-G1	LDM	Janthinobacterium	Gut	J2	106	15.45	8.54	3.49	0.146	118	0.90
LJ2-G2	LDM	Janthinobacterium	Gut	J2	67	9.72	8.02	5.44	0.145	74	0.90
LJ2-S1	LDM	Janthinobacterium	Skin	J2	110	8.73	4.20	2.39	0.079	121	0.91
LJ2-S2	LDM	Janthinobacterium	Skin	J2	116	10.93	6.04	2.94	0.094	152	0.76
LJ2-W	LDM	Janthinobacterium	Water	J2	193	13.71	7.91	4.37	0.071	324	0.60
LJ3-G2	LDM	Janthinobacterium	Gut	J3	58	2.26	1.38	1.18	0.039	65	0.90
LJ3-G3	LDM	Janthinobacterium	Gut	J3	65	1.85	1.24	1.11	0.028	88	0.74
LJ3-S1	LDM	Janthinobacterium	Skin	J3	143	10.08	4.46	2.96	0.070	158	0.91
LJ3-S3	LDM	Janthinobacterium	Skin	J3	123	6.74	3.47	2.56	0.055	135	0.91
LJ3-W	LDM	Janthinobacterium	Water	J3	133	7.09	3.60	2.17	0.053	149	0.90
LU1-G1	LDM	Untreated	Gut	U1	113	9.23	4.25	2.26	0.082	129	0.88
LU1-G2	LDM	Untreated	Gut	U1	147	7.67	3.39	2.04	0.052	151	0.97
LU1-G3	LDM	Untreated	Gut	U1	98	6.07	3.05	1.91	0.062	109	0.90
LU1-S1	LDM	Untreated	Skin	U1	119	6.44	3.18	1.97	0.054	133	0.90
LU1-S2	LDM	Untreated	Skin	U1	147	9.65	4.50	2.66	0.066	161	0.92
LU1-S3	LDM	Untreated	Skin	U1	143	10.29	4.97	2.75	0.072	169	0.85

LU1-W	LDM	Untreated	Water	U1	145	7.86	3.45	1.97	0.054	164	0.89
LU2-G1	LDM	Untreated	Gut	U2	38	2.85	2.00	1.50	0.075	40	0.95
LU2-G2	LDM	Untreated	Gut	U2	77	3.00	1.76	1.35	0.039	95	0.81
LU2-S1	LDM	Untreated	Skin	U2	133	7.64	4.04	2.55	0.057	167	0.80
LU2-S2	LDM	Untreated	Skin	U2	119	8.65	5.10	3.34	0.073	125	0.95
LU2-W	LDM	Untreated	Water	U2	173	15.87	8.83	4.11	0.092	197	0.88
LU3-G1	LDM	Untreated	Gut	U3	77	6.00	3.21	1.96	0.078	89	0.87
LU3-G2	LDM	Untreated	Gut	U3	28	1.02	1.00	1.00	0.036	35	0.79
LU3-S1	LDM	Untreated	Skin	U3	57	2.75	1.92	1.46	0.048	71	0.80
LU3-S2	LDM	Untreated	Skin	U3	59	3.42	2.03	1.47	0.058	113	0.52
LU3-W	LDM	Untreated	Water	U3	132	6.26	3.03	1.87	0.047	169	0.78
LY1-G1	LDM	Yersinia	Gut	Y1	31	1.26	1.07	1.04	0.041	45	0.69
LY1-G2	LDM	Yersinia	Gut	Y1	74	6.53	3.36	1.98	0.088	77	0.97
LY1-G3	LDM	Yersinia	Gut	Y1	72	3.70	1.96	1.43	0.051	86	0.84
LY1-S1	LDM	Yersinia	Skin	Y1	138	7.47	3.73	2.27	0.054	187	0.74
LY1-S2	LDM	Yersinia	Skin	Y1	97	6.23	3.60	2.66	0.064	106	0.92
LY1-S3	LDM	Yersinia	Skin	Y1	85	3.10	2.16	1.63	0.037	95	0.89
LY1-W	LDM	Yersinia	Water	Y1	175	7.90	3.29	1.90	0.045	190	0.92
LY2-G1	LDM	Yersinia	Gut	Y2	121	5.58	3.23	2.00	0.046	145	0.83
LY2-G3	LDM	Yersinia	Gut	Y2	86	7.49	4.15	2.36	0.087	92	0.94
LY2-S1	LDM	Yersinia	Skin	Y2	143	7.08	3.37	2.04	0.050	202	0.71
LY2-S3	LDM	Yersinia	Skin	Y2	84	3.42	2.47	2.10	0.041	385	0.22
LY2-W	LDM	Yersinia	Water	Y2	167	7.24	3.17	1.88	0.043	199	0.84

LY3-G1	LDM	Yersinia	Gut	Y3	115	8.34	4.60	2.94	0.073	122	0.95
LY3-G2	LDM	Yersinia	Gut	Y3	74	1.98	1.26	1.12	0.027	84	0.88
LY3-G3	LDM	Yersinia	Gut	Y3	28	1.09	1.02	1.01	0.039	38	0.74
LY3-S1	LDM	Yersinia	Skin	Y3	113	6.87	3.44	2.20	0.061	117	0.97
LY3-S2	LDM	Yersinia	Skin	Y3	125	5.03	2.38	1.62	0.040	145	0.86
LY3-S3	LDM	Yersinia	Skin	Y3	128	5.69	2.48	1.63	0.044	137	0.94
LY3-W	LDM	Yersinia	Water	Y3	196	9.86	4.94	2.82	0.050	240	0.82

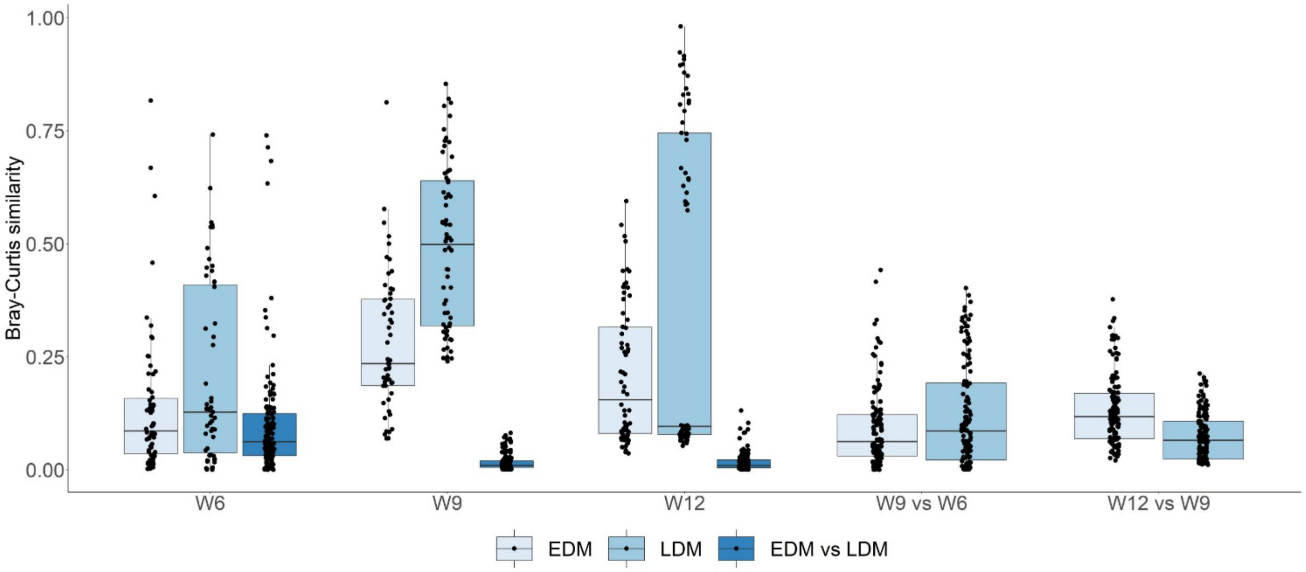

Supplementary Figure 1. Boxplot of the Bray-Curtis similarities of all samples from Exp.1. EDM and LDM samples are compared for each sampling timepoint. Represented as box in the boxplot are the median as well as the upper and lower quartile.

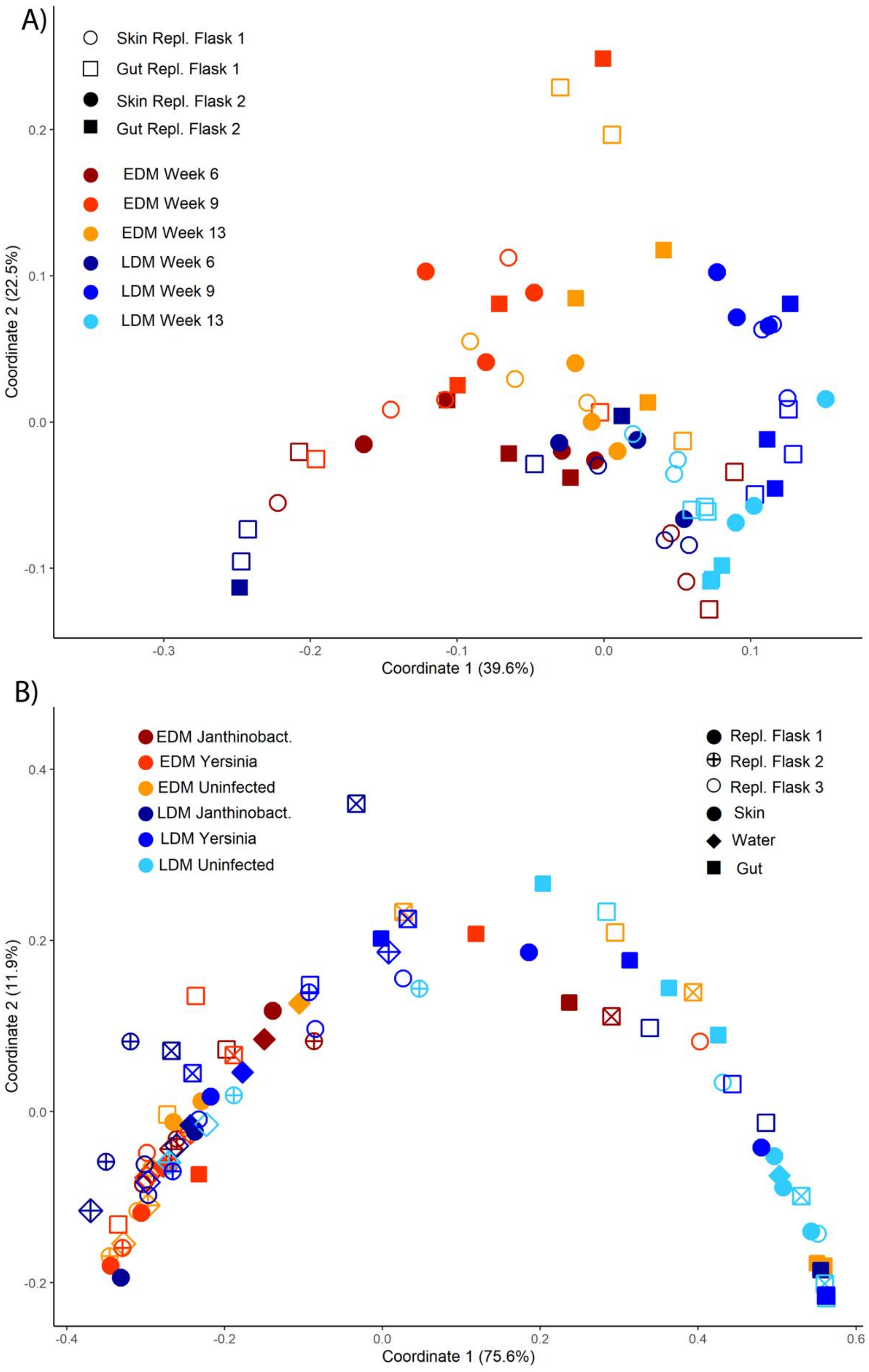

Supplementary Figure 2. PCoA of the weighted Uni-Frac analysis of all samples of Exp.1 (A) and Exp.2 (B).

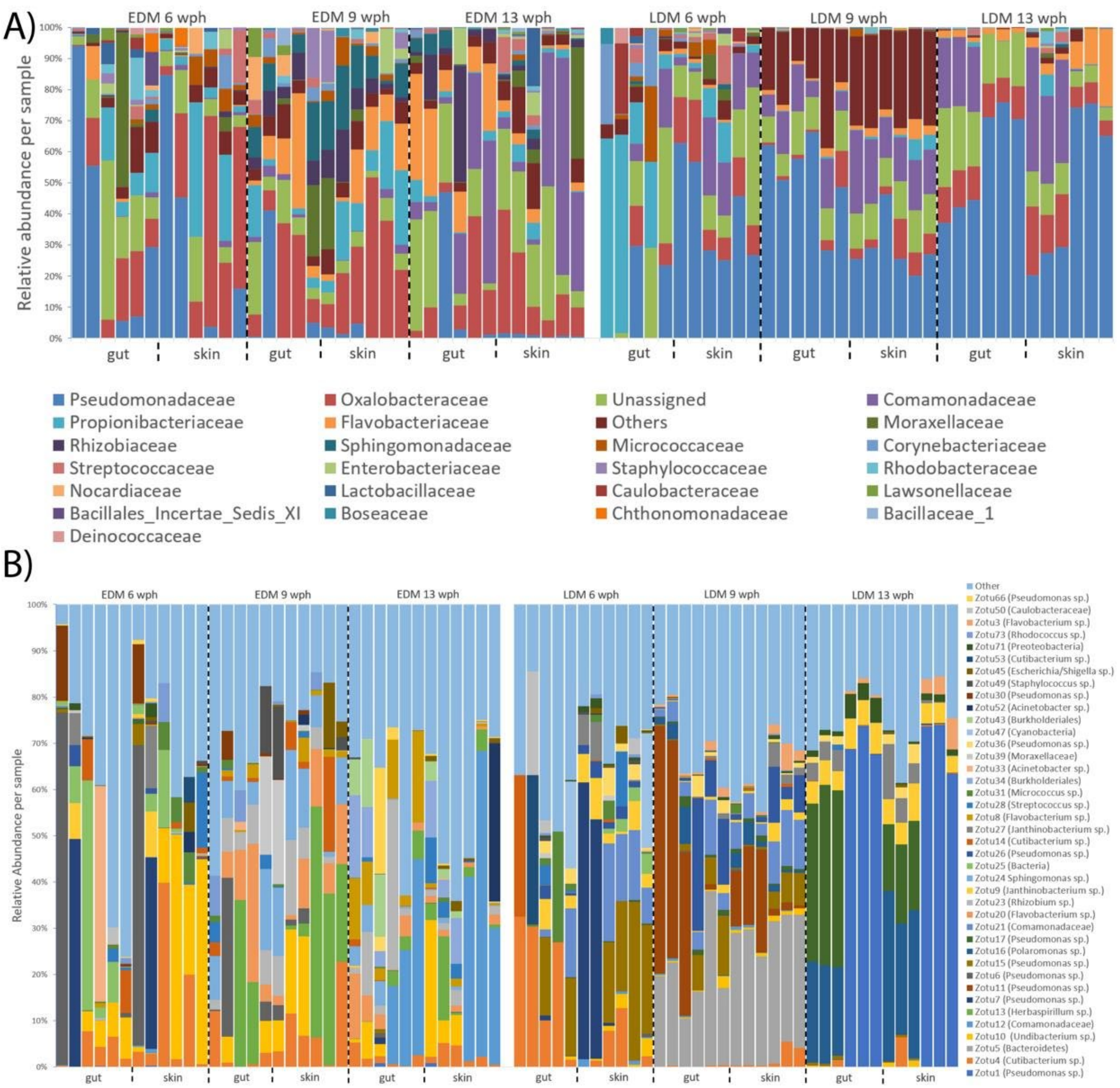

Supplementary Figure 3. Community composition of all samples from Exp.1 at the family level (A) and the ASV level (B). A) For each phylum, the four most abundant families are shown, others are summarized as "others". ASVs not classified at family level are shown at the highest taxonomic level B) The 40 most abundant ASVs are shown until their lowest taxonomic level, the others are summarized as "Others".

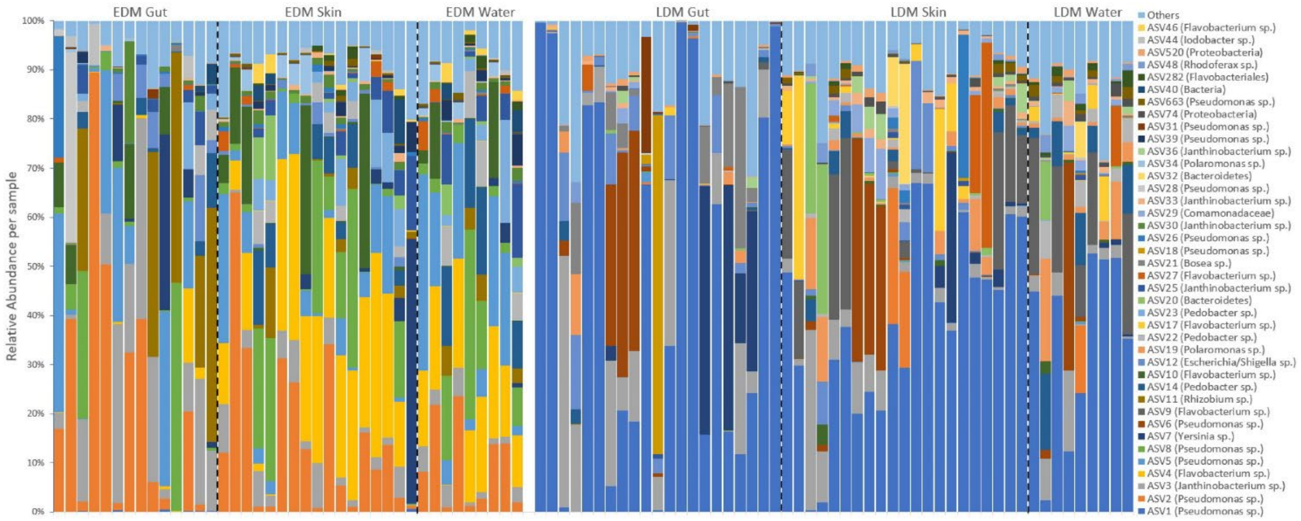

Supplementary Figure 4. The bacterial compositions of all samples of Exp.2 at the ASV level. The 40 most abundant ASVs are shown until their lowest taxonomic level. The samples are sorted according to EDM and LDM samples and further according to their sample type.

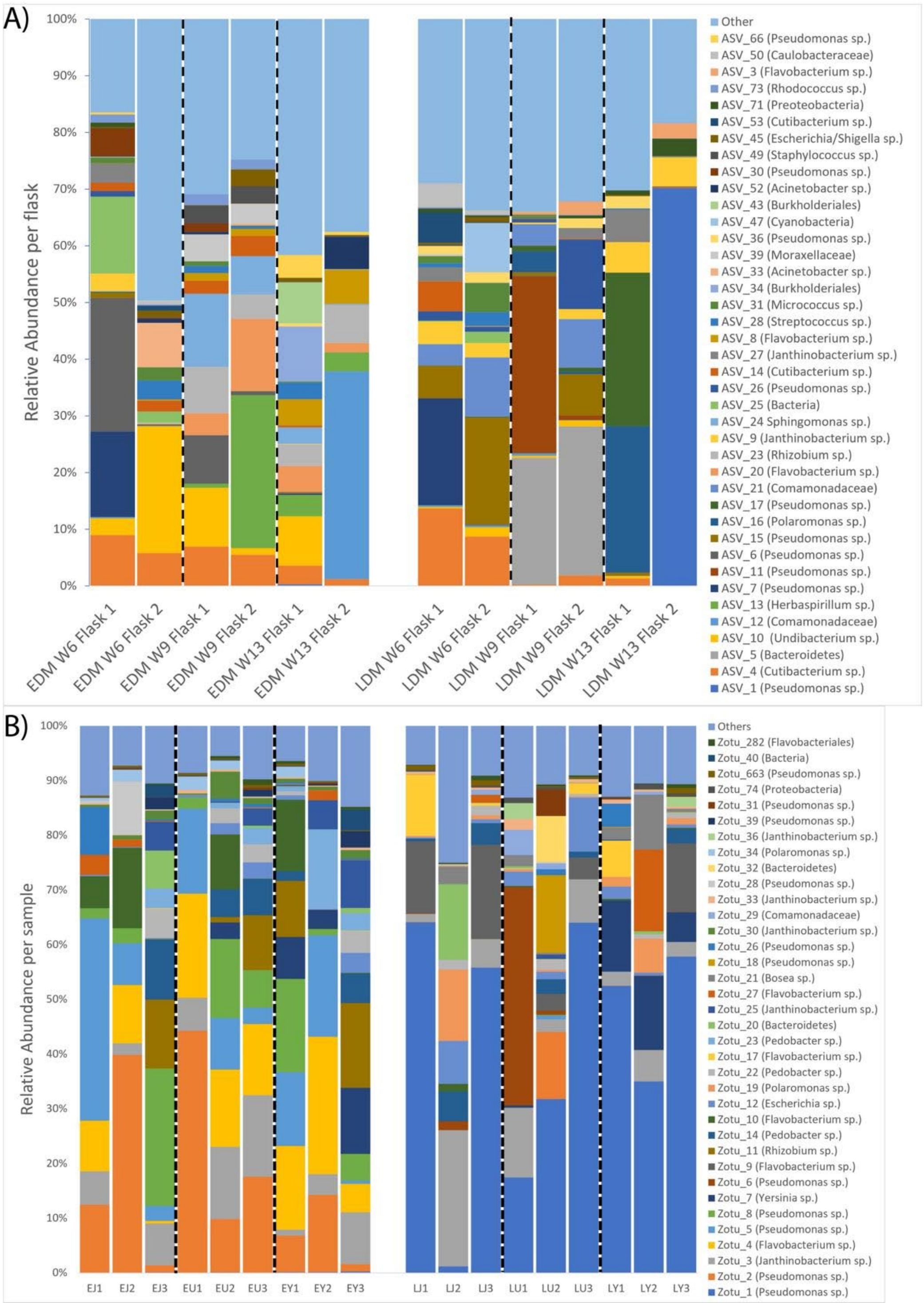

Supplementary Figure 5. Average bacterial composition of all flasks (both gut and skin samples) of Exp.1 (A) and Exp.2 (B) on ASV level.

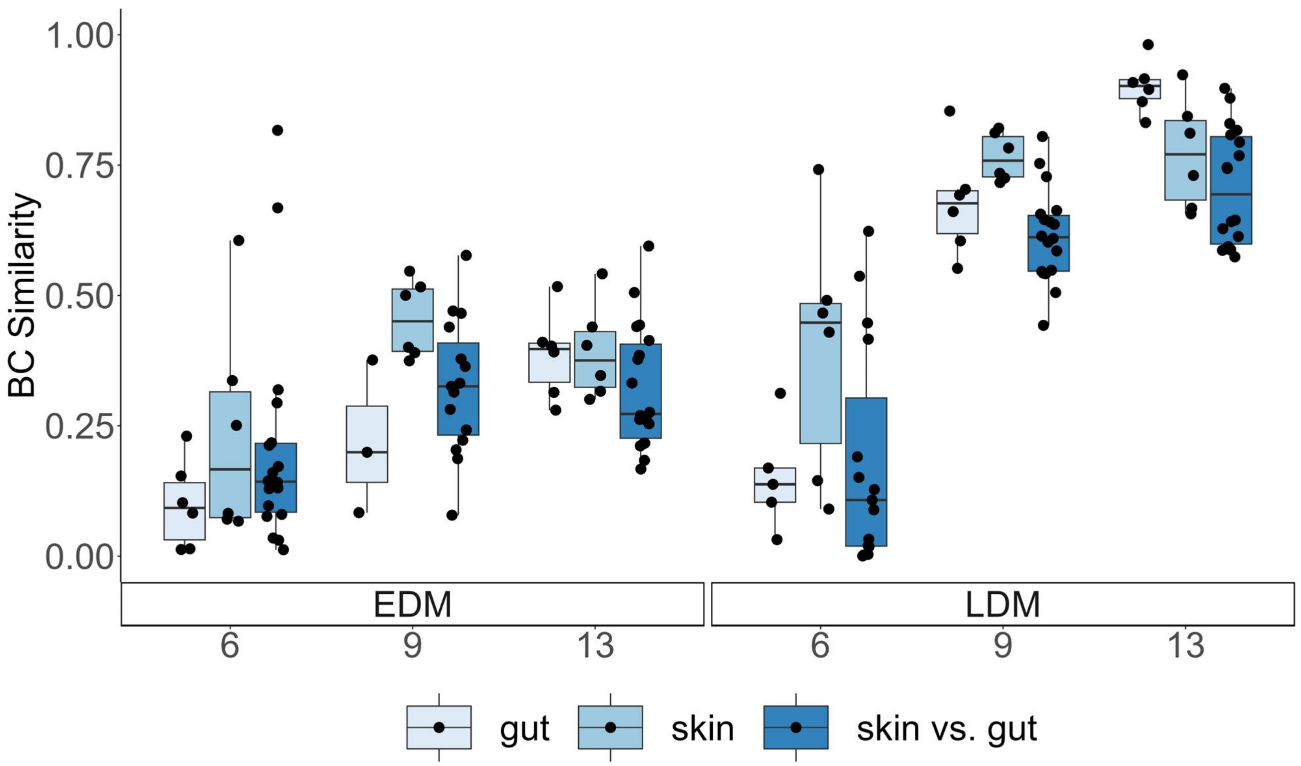

Supplementary Figure 6. Boxplot of the Bray-Curtis similarities of all samples from Exp.1. Within each sampling flask and timepoint, all three skin and gut samples are both compared within (gut; skin) and between each other (skin vs. gut). Represented as box in the boxplot are the median as well as the upper and lower quartile.

A)

5’-
ACATGCAAGTCGAGCGGCAGCGGAAAGTAGCTTGCTACTTTGCCGGCGAGCGGCGGAC
GGGTGAGTAATGTCTGGGGATCTGCCTGATGGAGGGGGATAACTACTGGAAACGGTAGC
TAATACCGCATAACCTCGCAAGAGCAAAGTGGGGGACCTTCGGGCCTCACGCCATCGGA
TGAACCCAGATGGGATTAGCTAGTAGGTGGGGTAATGGCTCACCTAGGCGACGATCCCT
AGCTGGTCTGAGAGGATGACCAGCCACACTGGAACTGAGACACGGTCCAGACTCCTACG
GGAGGCAGCAGTGGGGAATATTGCACAATGGGCGCAAGCCTGATGCAGCCATGCCGCG
TGTGTGAAGAAGGCCTTCGGGTTGTAAAGCACTTTCAGCGAGGAGGAAGGGTTAAGTGT
TAATAGCACTGAACATTGACGTTACTCGCAGAAGAAGCACCGGCTAACTCCGTGCCAGC
AGCCGCGGTAATACGGAGGGTGCAAGCGTTAATCGGAATTACTGGGCGTAAAGCGCAC
GCAGGCGGTTTGTTAAGTCAGATGTGAAATCCCCGAGCTTAACTTGGGAACTGCATTTG
AAACTGGCAAGCTAGAGTCTTGTAGAGGGGGGTAGAATTCCAGGTGTAGCGGTGAAAT
GCGTAGAGATCTGGAGGAATACCGGTGGCGAAGGCGGCCCCCTGGACAAAGACTGACG
CTCAGGTGCGAAAGCGTGGGGAGCAAACAGGATTAGATACCCTGGTAGTCCACGCTGTA
AACGATGTCGACTTGGAGGTTGTGCCCTTGAGGCGTGGCTTCCGGAGCTAACGCGTTAA
GTCGACCGCCTGGGGAGTACGGCCGCAAGGTTAAAACTCAAATGAATTGACGGGGGCC
CGCACAAGCGGTGGAGCATGTGGTTTAATTCGATGCAACGCGAAGAACCTTACCTACTC
TTGACATCCACAGAACTTGGCAGAGATGCCTTGGTGCCTTCGGGAACTGTGAGACAGGT
GCTGCATGGCTGTCGTCAGCTCGTGTTGTGAAATGTTGGGTTAAGTCCCGCAACGAGCG
CAACCCTTATCCTTTGTTGCCAGCACGTAATGGTGGGAACTCAAGGGAGACTGCCGGTG
ACAAACCGGAGGAAGGTGGGGATGACGTCAAGTCATCATGGCCCTTACGAGTAGGGCT
ACACACGTGCTACAATGGCAGATACAAAGTGAAGCGAACTCGCGAGAGCAAGCGGACC
ACATAAAGTCTGTCGTAGTCCGGATTGGAGTCTGCAACTCGACTCCATGAAGTCGGAAT
CGCTAGTAATCGTAGATCAGAATGCTACGGTGAATACGTTCCCGGGCCTTGTACACACC
GCCCGTCACACCATGGGAGTGGGTTGCAAAAGAAGTAGGTAGCTTAACCTTCGGGAGGG
CGCTTA-3’

B)

5’-
CATGCAAGTCGAACGGCAGCACGGAGCTTGCTCTGGTGGCGAGTGGCGAACGGGTGAG

TAATATATCGGAACGTACCCTGGAGTGGGGGATAACGTAGCGAAAGTTACGCTAATACC
GCATACGATCTAAGGATGAAAGTGGGGGATCGCAAGACCTCATGCTCGTGGAGCGGCC
GATATCTGATTAGCTAGTTGGTAGGGTAAAAGCCTACCAAGGCATCGATCAGTAGCTGG
TCTGAGAGGACGACCAGCCACACTGGAACTGAGACACGGTCCAGACTCCTACGGGAGG
CAGCAGTGGGGAATTTTGGACAATGGGCGAAAGCCTGATCCAGCAATGCCGCGTGAGTG
AAGAAGGCCTTCGGGTTGTAAAGCTCT**Y**TTGTCAGGGAAGAAACGGTGAGAGCTAATAT
CTCTTGCTAATGACGGTACCTGAAGAATAAGCACCGGCTAACTACGTGCCAGCAGCCGC
GGTAATACGTAGGGTGCAAGCGTTAATCGGAATTACTGGGCGTAAAGCGTGCGCAGGCG
GTTTTGTAAGTCTGATGTGAAATCCCCGGGCTCAACCTGGGAATTGCATTGGAGACTGC
AAGGCTAGAATCTGGCAGAGGGGGGTAGAATTCCACGTGTAGCAGTGAAATGCGTAGA
TATGTGGAGGAACACCGATGGCGAAGGCAGCCCCCTGGGTCAAGATTGACGCTCATGCA
CGAAAGCGTGGGGAGCAAACAGGATTAGATACCCTGGTAGTCCACGCCCTAAACGATGT
CTACTAGTTGTCGGGTCTTAATTGACTTGGTAACGCAGCTAACGCGTGAAGTAGACCGC
CTGGGGAGTACGGTCGCAAGATTAAAACTCAAAGGAATTGACGGGGACCCGCACAAGC
GGTGGATGATGTGGATTAATTCGATGCAACGCGAAAAACCTTACCTACCCTTGACATGG
CTGGAATCCTCGAGAGATTGAGGAGTGCTCGAAAGAGAACCAGTACACAGGTGCTGCAT
GGCTGTCGTCAGCTCGTGTCGTGAGATGTTGGGTTAAGTCCCGCAACGAGCGCAACCCT
TGTCATTAGTTGCTACGAAAGGGCACTCTAATGAGACTGCCGGTGACAAACCGGAGGAA
GGTGGGGATGACGTCAAGTCCTCATGGCCCTTATGGGTAGGGCTTCACACGTCATACAA
TGGTACATACAGAGCGCCGCCAACCCGCGAGGGGGAGCTAATCGCAGAAAGTGTATCG
TAGTCCGGATTGTAGTCTGCAACTCGACTGCATGAAGTTGGAATCGCTAGTAATCGCGG
ATCAGCATGTCGCGGTGAATACGTTCCCGGGTCTTGTACACACCGCCCGTCACACCATG
GGAGCGGGTTTACCAGAAGTAGGTAGCTTAACCGCAAGGAGGGCGCTTACCAC-3’

Supplementary Figure 7. The partial 16S rRNA gene sequence of A) *Yersinia ruckeri* 06059 and B) *Janthinobacterium* sp. 3.108. These sequences were obtained by Sanger sequencing of the partial 16S rRNA gene using the primers Eub8F (5’- AGA GTT TGA TCM TGG CTC AG -3’) and 1492R (5’- GGT TAC CTT GTT ACG ACT T -3’). Note that *J*. sp. 3.108 has an ambiguous nucleotide position, for which signals for both C (corresponding to ASV3 in the dataset) and T (corresponding to ASV15 in the dataset) were observed (indicated by the letter Y).

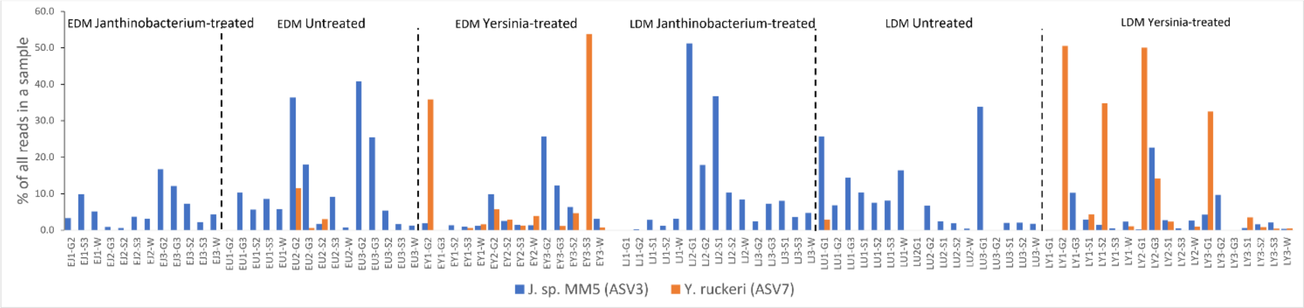

Supplementary Figure 8. Colonisation success of *Yersinia ruckeri* 06059 and *Janthinobacterium* sp. 3.108. For each sample, the relative amount of the ASV of each strain is given.

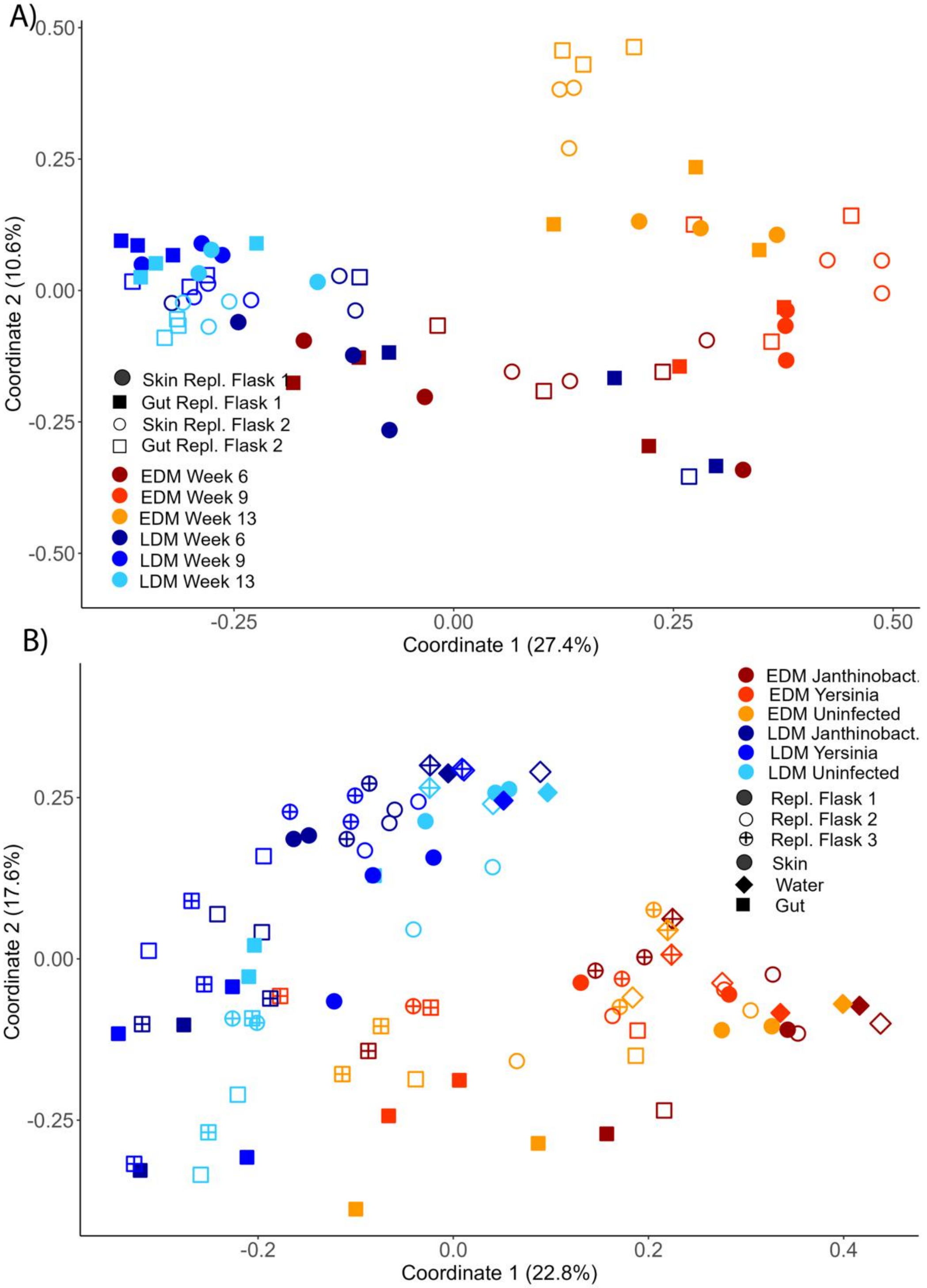

Supplementary Figure 9. PCoA of the Sørensen-Dice index of all samples of Exp.1 (A) and Exp.2 (B)
